# Global shifts in osteoarthritis subtype trends among older adults due to elevated BMI: an age-period-cohort analysis based on the global burden of disease database

**DOI:** 10.3389/fpubh.2025.1518572

**Published:** 2025-04-28

**Authors:** Zhiqiang Li, Yaxiang Chen, Zhao Shen

**Affiliations:** Department of Orthopedics, Cangzhou People’s Hospital, Cangzhou, Hebei, China

**Keywords:** osteoarthritis, disease burden, BMI, older adults, Sociodemographic status, GBD 2021

## Abstract

**Purpose:**

It remains unclear whether elevated Body Mass Index(BMI)has a similar impact on the disease burden of osteoarthritis subtypes in older adults. This study aims to compare the long-term trends of osteoarthritis subtypes caused by high BMI across different gender groups globally from 1990 to 2021.

**Methods:**

We obtained cross-sectional data from the Global Burden of Disease, Injuries, and Risk Factors Study (GBD) 2021 (https://vizhub.healthdata.org/gbd-results/). The disease burden of osteoarthritis subtypes in older adults attributable to high BMI was quantified using Years Lived with Disability (YLDs). Linear regression and the Age-Period-Cohort (APC) method were employed to calculate the trends in Age-standardized Years lived with disability rate (ASYR), adjusting for age, period, and cohort effects.

**Results:**

The ASYR of osteoarthritis attributable to high BMI in older adults globally has shown a continuous upward trend over the past 32 years, with an Estimated Average Percentage Change (EAPC) of 0.96 (95% CI: 0.94 to 0.99). Specifically, the EAPC for hip osteoarthritis was 0.73 (95% CI: 0.70 to 0.76), while for knee osteoarthritis, it was 0.99 (95% CI: 0.96 to 1.02). China recorded the highest number of osteoarthritis YLDs globally, reaching 0.59 million (95% UI: −0.05 to 1.71). The United States had one of the highest ASYR rates for osteoarthritis at 410.85 per 100,000 (95% UI: −44.47 to 1,083.52), while India exhibited the highest EAPC for osteoarthritis worldwide at 2.74 (95% CI: 2.70 to 2.79), with hip osteoarthritis at 3.36 (95% CI: 3.25 to 3.48) and knee osteoarthritis at 2.70 (95% CI: 2.65 to 2.75). The local drift curves indicated a slow upward trend in the annual percentage change of YLDs for both hip and knee osteoarthritis attributable to high BMI across all age groups. In terms of gender, males exhibited a higher rate and risk of YLDs associated with high BMI.

**Conclusion:**

Our findings provide strong evidence that the ASYR associated with high BMI globally have continuously increased over the past 32 years, with consistent patterns of change observed across different osteoarthritis subtypes. This highlights the critical role of BMI control in effectively alleviating the burden of osteoarthritis in older adults.

## Introduction

Osteoarthritis is one of the most prevalent chronic musculoskeletal diseases among older adults globally, leading to long-term pain, functional impairment, and a decline in quality of life (1–3). With the global rise in ageing and obesity rates, the burden of osteoarthritis is expected to continue to increase. High BMI is widely recognised as a major risk factor for osteoarthritis, particularly in weight-bearing joints such as the knee and hip (4). According to findings from the GBD 2021 study, obesity is one of the top five modifiable health risk factors worldwide, and the contribution of high BMI to the osteoarthritis burden has become increasingly evident over the past few decades (5).

Although the association between high BMI and osteoarthritis is well-established, previous studies, including those from the GBD project, have yet to provide a comprehensive estimate of the long-term impact of high BMI on osteoarthritis and its subtypes (6, 7). Most existing research has focused solely on the overall burden of osteoarthritis, without thoroughly exploring the differences between osteoarthritis subtypes in different anatomical sites, such as knee osteoarthritis and hip osteoarthritis, nor considering the impact of high BMI across different age groups (8–10). Therefore, assessing the burden trends of various osteoarthritis subtypes caused by high BMI is crucial for developing targeted prevention strategies.

In this study, we utilised data obtained from the GBD 2021 database to describe the YLDs due to osteoarthritis and its subtypes attributable to high BMI. We calculated the EAPC to reveal the global trends in the burden of osteoarthritis subtypes in older adults caused by high BMI from 1990 to 2021. Additionally, we employed an APC model to analyse the effects of age, period, and cohort on the prevalence of osteoarthritis subtypes.

## Methods

### Study population and data collection

The data for this study were sourced from the GBD 2021 dataset, which provides comprehensive information on the global and regional burden of 371 diseases and injuries, along with 88 risk factors, across 204 countries and territories from 1990 to 2021. The data was accessed through the Global Health Data Exchange (GHDx) platform,^1^ where detailed information on the disease burden indicators, including YLDs for osteoarthritis in older adults aged 60 and above, was downloaded. The dataset also provides data on various demographic groups, osteoarthritis subtypes (hip and knee osteoarthritis), and BMI-related factors. According to the World Health Organization (WHO) standards, high BMI is defined as a BMI of 25 kg/m^2^ or higher, which includes overweight (BMI 25–29.9 kg/m^2^) and obesity (BMI ≥ 30 kg/m^2^) (11). In contrast to the WHO definition, which applies to all age groups, the GBD group uses different definitions for children and adults based on specific age cutoffs. In this study, we applied the GBD-specific definition of high BMI, as outlined in the GBD methods (source: https://www.healthdata.org/sites/default/files/methods_appendices/2021/Cogen_metab_bmi_writeup_gbd2020_updated01-31%E2%80%932024.pdf). Data on socio-demographic index (SDI) were also collected to assess the impact of socioeconomic factors, which could provide a context for understanding the influence of regional disparities on the disease burden. As this study utilised a publicly available database containing de-identified data, ethical approval was not required. This study adhered to the Guidelines for Accurate and Transparent Health Estimates Reporting (GATHER) (12).

### Statistical analysis

To examine the global distribution, regional disparities, and population-level status of the osteoarthritis burden and its subtypes among older adults, we quantified the burden attributable to high BMI using the ASYR obtained from the GBD 2021 dataset. Global mapping and regional comparative analyses were conducted to visualise the distribution of osteoarthritis burden. Data were aggregated according to the geographical regions defined by the GBD study, with maps created using R (version 4.3.2) and the ‘ggplot2’ and ‘sf’ packages. Linear regression was chosen as a statistical method due to its effectiveness in modeling continuous data trends over time, which allows for the evaluation of changes in osteoarthritis burden in relation to BMI over the 32-year period. The APC model was specifically employed because it accounts for the impact of age, period, and cohort effects independently, making it suitable for analyzing long-term trends influenced by various temporal factors, such as population aging, medical advancements, and societal changes [38913425]. The EAPC was employed to assess the time trends of ASYR from 1990 to 2021, with EAPC values calculated through linear regression models and outputs processed using the ‘broom’ package. This method is widely used for studying disease burden trends and has been validated in previous studies for its capacity to quantify annual percentage changes in disease rates [39,435,408, 38,745,964].

To eliminate the impact of differences in population age structure across regions or time periods on disease burden, this study uses the direct standardization method to calculate the Age-Standardized Rate (ASR). The specific age group rate data is selected, and the age categories from the GBD database are matched with the global standard population data to obtain the standard population weights for each age group. The weighted average rate is then calculated using the following formula: (Val is the raw rate value for the specific age group, Population_weight is the standard population weight for that age group).


$$ASR=\frac{\sum \left(\mathrm{val}\times \mathrm{Population}\_\mathrm{weight}\right)}{\sum \left(\mathrm{Population}\_\mathrm{weight}\right)}$$

The APC model was employed to assess the impact of age, period, and cohort on YLD rates for osteoarthritis subtypes attributable to high BMI. APC analysis minimises the interaction effects among these three factors, providing more precise estimates for each. The age effect reflects the influence of population ageing, while the period effect captures changes in YLD rates over time that affect all age groups, often due to advancements in disease screening, medical technologies, or reclassification of diseases. The cohort effect highlights long-term trends in disease incidence and mortality, shaped by cohort-specific lifestyles, environmental changes, and exposure to risk factors (13, 14).

In this study, within the APC framework, we evaluated the following parameters: net drift, which represents the overall annual percentage change in the attributable YLD rate; local drift, indicating the annual percentage change in YLD rates for each age group; the longitudinal age curve, which reflects the age effect by showing age-specific rates after adjusting for drift; period rate ratios (PRR), which represent the period effect and the relative risk of YLDs across different time periods; and cohort rate ratios (CRR), which reflect the cohort effect and the relative risk of YLDs across different birth cohorts. The APC analysis considered age groups starting from 60 years, segmented into 5-year intervals (e.g., 60–64 years, 65–69 years, etc.). Trends were assessed for statistical significance using 95% confidence intervals (CI), and descriptive statistics for all key variables were presented as mean values with 95% uncertainty intervals (UI). All analyses were conducted using R version 4.3.2.

## Results

### Global and SDI regional trends in YLDs

**Figure 1:**
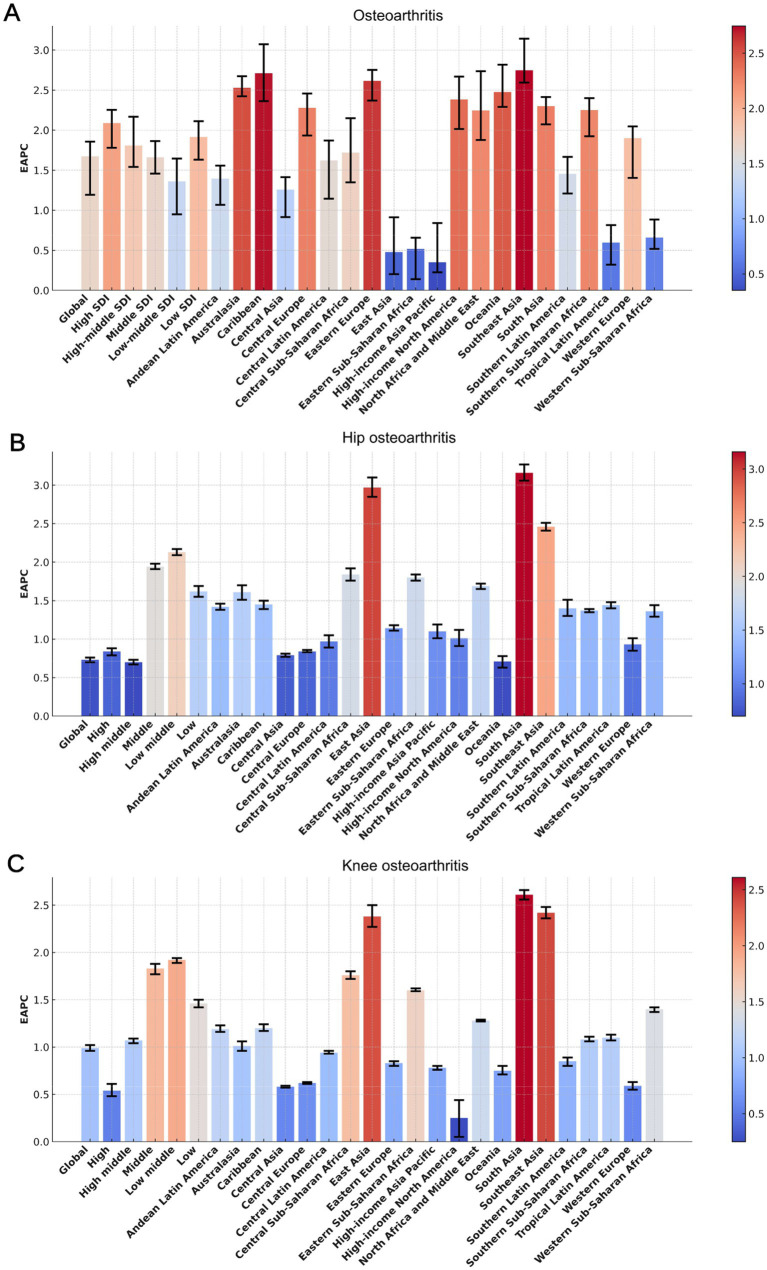
The EAPC of the ASYR for osteoarthritis **(A)**, hip osteoarthritis **(B)**, and knee osteoarthritis **(C)** among individuals aged 60 years and above, stratified by GBD regions and SDI quintiles, from 1990 to 2021. EAPC, Estimated Average Percentage Changes; GBD, Global Burden of Disease; SDI, Socio-demographic Index; ASYR, Age-standardized Years lived with disability rate.

**Figure 2:**
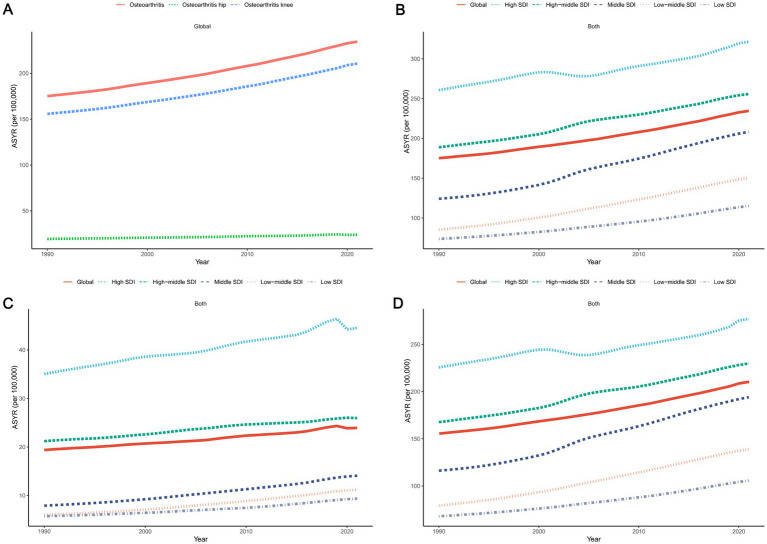
The trends in osteoarthritis, hip osteoarthritis, and knee osteoarthritis among individuals aged 60 years and above from 1990 to 2021 **(A)**, as well as the trends for osteoarthritis **(B)**, hip osteoarthritis **(C)**, and knee osteoarthritis **(D)** classified by the SDI quintiles. ASYR, Age-standardized Years lived with disability rate; YLDs, Years Lived with Disability; SDI, Socio-demographic Index.

In 1990, the YLDs attributable to high BMI for osteoarthritis in older adults globally were 84.02 ten thousands (95% UI: −7.23 to 240.44), corresponding to an ASYR of 175.06 per 100,000 (95% UI: −14.94 to 500.89). By 2021, the global YLDs had significantly increased to 2.55 million (95% UI: −0.24 to 7.18), with the ASYR rising to 234.51 per 100,000 (95% UI: −21.52 to 659.64). Although the 95% UI for YLDs and ASYR include negative values, it is important to note that the presence of these negative intervals reflects the inherent uncertainty in the data and does not invalidate the overall trends. These intervals are a statistical artifact of the model’s estimation process, particularly when estimating changes over long periods across diverse global regions. The EAPC was 0.96 (95% CI: 0.94 to 0.99). While the 95% CI for the EAPC is narrow, indicating statistical significance, it is essential to acknowledge that the UI ranges for YLDs and ASYR suggest that the estimates are subject to uncertainty, which should be considered when interpreting the results. The YLDs for hip osteoarthritis increased from 9.04 ten thousands (95% UI: −0.81 to 24.50) in 1990 to 25.82 (95% UI: −2.44 to 68.07) in 2021. The global ASYR for hip osteoarthritis rose from 19.37 per 100,000 (95% UI: −1.71 to 52.62) in 1990 to 23.93 per 100,000 (95% UI: −2.25 to 63.13) in 2021, with an EAPC of 0.73 (95% CI: 0.70 to 0.76). The YLDs for knee osteoarthritis increased significantly from 0.75 million (95% UI: −0.64 to 2.15) in 1990 to 2.30 million (95% UI: −0.21 to 6.46) in 2021. In 1990, the global ASYR for knee osteoarthritis was 155.69 per 100,000 (95% UI: −13.22 to 446.14), rising to 210.57 per 100,000 (95% UI: −19.24 to 593.06) by 2021. The EAPC for ASYR of knee osteoarthritis was 0.99 (95% CI: 0.96 to 1.02), showing a growth trend closely aligned with overall osteoarthritis, slightly higher than the growth rate for hip osteoarthritis (Figures 1A-C, 2A, Table 1).

**Table 1:** The global YLDs and ASYR, as well as those of the SDI regions and 21 specific regions in 1990 and 2021, along with the EAPC of ASYR from 1990 to 2021.

	Osteoarthritis					Osteoarthritis hip					Osteoarthritis knee				
	1990		2021		EAPC(95% CI)	1990		2021		EAPC(95% CI)	1990		2021		EAPC(95% CI)
	No (95% UI)	ASR (per 100,000)	No (95% UI)	ASR (per 100,000)		No (95% UI)	ASR (per 100,000)	No (95% UI)	ASR (per 100,000)		No (95% UI)	ASR (per 100,000)	No (95% UI)	ASR (per 100,000)	
Global	840216.56 (-72282.49,2404438.07)	175.06 (-14.94,500.89)	2553595.45 (-235000.8,7182421.69)	234.51 (-21.52,659.64)	0.96 (0.94,0.99)	90413.92 (-8083.43,244926.06)	19.37 (-1.71,52.62)	258221.45 (-24406.87,680696.42)	23.93 (-2.25,63.13)	0.73 (0.70,0.76)	749802.64 (-64163.74,2148107.4)	155.69 (-13.22,446.14)	2295374 (-210359.29,6461681.93)	210.57 (-19.24,593.06)	0.99 (0.96,1.02)
Sex															
Male	276719.32 (-23903.58,794419.08)	129.18 (-11.00,371.33)	908210.68 (-84294.04,2560489.18)	181.04 (-16.67,510.43)	1.11 (1.08,1.13)	35013.44 (-3124.75,95236.06)	16.97 (-1.49,46.39)	111283.29 (-10558.44,295569.83)	22.65 (-2.13,60.24)	0.98 (0.96,1.01)	241705.88 (-20773.93,700281.23)	112.21 (-9.51,325.50)	796927.39 (-73580.16,2258527.55)	158.39 (-14.52,448.94)	1.13 (1.10,1.15)
Female	563497.24 (-48441.11,1610367.59)	211.05 (-18.09,603.09)	1645384.77 (-150706.76,4613028.47)	280.59 (-25.72,786.43)	0.94 (0.92,0.97)	55400.48 (-4952.49,150041.59)	21.11 (-1.88,57.28)	146938.16 (-13848.57,386085.28)	25.01 (-2.36,65.70)	0.61 (0.57,0.64)	508096.76 (-43448.22,1447917.84)	189.94 (-16.19,541.39)	1498446.61 (-136818.83,4204663.91)	255.58 (-23.35,717.09)	0.98 (0.95,1.01)
SDI quintile															
High	377303.18 (-33997.35,1056282.59)	260.65 (-23.51,729.54)	880838.81 (-85441.11,2393556.87)	321.54 (-31.53,872.93)	0.58 (0.53,0.64)	50700.77 (-4742.39,139532.21)	35.05 (-3.28,96.44)	122935.61 (-12291.82,322603.63)	44.51 (-4.49,116.59)	0.84 (0.79,0.88)	326602.41 (-29187.84,918030.22)	225.60 (-20.19,634.20)	757903.2 (-72951.06,2082554.95)	277.03 (-26.95,760.27)	0.54 (0.48,0.61)
High middle	234997.1 (-20702.85,666602.55)	188.80 (-16.48,536.42)	656472.82 (-62929.52,1811140.61)	255.78 (-24.47,705.47)	1.03 (1.00,1.05)	25395.87 (-2227.75,68492.62)	21.20 (-1.84,57.32)	65927.81 (-6341.78,174469.21)	25.95 (-2.49,68.67)	0.70 (0.67,0.73)	209601.23 (-18434.04,594772.63)	167.60 (-14.61,476.18)	590545.01 (-56565.87,1637994.66)	229.83 (-21.97,637.34)	1.07 (1.04,1.09)
Middle	149041.6 (-12027.25,440339.99)	124.08 (-9.90,365.78)	691041.8 (-60519.08,1990584.66)	208.12 (-18.11,599.89)	1.83 (1.78,1.89)	8871.15 (-702.53,24406.87)	7.90 (-0.62,21.79)	45445.95 (-3917.79,122689.49)	14.06 (-1.20,38.07)	1.94 (1.91,1.98)	140170.46 (-11323.92,415148.67)	116.18 (-9.28,343.46)	645595.85 (-56596.74,1856847.46)	194.06 (-16.91,558.58)	1.83 (1.77,1.88)
Low middle	58750.56 (-4568.55,175354.78)	85.20 (-6.56,253.99)	257084.85 (-21550.37,742464.92)	149.98 (-12.45,433.31)	1.93 (1.91,1.96)	3923.56 (-299.41,10952.06)	5.98 (-0.45,16.68)	18538.86 (-1497.85,50342.69)	11.17 (-0.89,30.39)	2.13 (2.09,2.17)	54826.99 (-4268.78,163657.61)	79.21 (-6.10,236.10)	238545.98 (-20040.35,688272.53)	138.81 (-11.55,400.16)	1.92 (1.89,1.94)
Low	19012.47 (-1374.26,58564.63)	73.63 (-5.29,226.41)	65703.12 (-4860.35,197663.42)	115.02 (-8.42,345.96)	1.48 (1.43,1.52)	1392.28 (-98.92,3991.4)	5.72 (-0.40,16.36)	5081.84 (-369.04,14253.41)	9.34 (-0.67,26.26)	1.62 (1.55,1.69)	17620.2 (-1280.51,54278.54)	67.91 (-4.90,208.95)	60621.28 (-4492.78,181898.33)	105.68 (-7.75,317.04)	1.46 (1.42,1.50)
GBD region															
Andean Latin America	5003.51 (-444.56,14167.02)	210.44 (-18.65,596.83)	21791.43 (-2179.65,59409.88)	302.52 (-30.14,825.32)	1.21 (1.17,1.24)	344.31 (-29.85,943.05)	14.73 (-1.27,40.37)	1621.7 (-153.22,4326.83)	22.65 (-2.13,60.46)	1.42 (1.38,1.46)	4659.19 (-413.4,13240.49)	195.71 (-17.32,557.06)	20169.74 (-2024.65,54884.84)	279.87 (-27.98,762.03)	1.19 (1.16,1.23)
Australasia	8534.11 (-808.37,23879.1)	274.13 (-26.01,767.14)	26940.1 (-2674.53,72041.52)	384.01 (-38.30,1027.56)	1.09 (1.04,1.15)	1035.29 (-98.95,2824.81)	33.34 (-3.19,90.95)	3822.79 (-384.75,10188.69)	54.16 (-5.48,144.40)	1.61 (1.51,1.70)	7498.82 (-707.23,20848.16)	240.79 (-22.75,669.59)	23117.31 (-2285.01,62298.96)	329.85 (-32.74,890.47)	1.01 (0.96,1.06)
Caribbean	6301.46 (-529.16,18112.72)	195.71 (-16.41,562.74)	18734.92 (-1771.6,52029.71)	279.01 (-26.40,774.97)	1.22 (1.18,1.26)	453.08 (-37.35,1234.49)	14.29 (-1.18,38.98)	1451.29 (-131.92,3827.07)	21.50 (-1.96,56.69)	1.45 (1.39,1.50)	5848.39 (-492.12,16707.77)	181.42 (-15.24,518.40)	17283.63 (-1635.8,48079.38)	257.51 (-24.39,716.49)	1.20 (1.17,1.24)
Central Asia	9483.92 (-854.34,26541.44)	170.72 (-15.16,479.43)	19725.25 (-2014.76,53394.55)	205.77 (-20.36,559.69)	0.61 (0.60,0.62)	1324.77 (-115.96,3598.82)	24.56 (-2.12,66.93)	2864.54 (-277.8,7573.27)	31.28 (-2.95,83.10)	0.79 (0.77,0.81)	8159.15 (-733.69,22926.65)	146.16 (-12.97,411.57)	16860.71 (-1726.71,45541.86)	174.49 (-17.33,472.09)	0.58 (0.57,0.59)
Central Europe	40542.04 (-3695.9,111801.16)	209.57 (-19.02,579.35)	77025.47 (-7840.05,207185.17)	255.02 (-26.09,685.90)	0.65 (0.64,0.66)	5332 (-474.74,14171.15)	28.72 (-2.54,76.64)	11170.67 (-1093.79,29181.68)	36.79 (-3.62,96.03)	0.84 (0.83,0.86)	35210.04 (-3218.64,97086.81)	180.85 (-16.47,499.65)	65854.8 (-6733.51,177826.64)	218.23 (-22.43,589.29)	0.62 (0.61,0.63)
Central Latin America	22761.11 (-2090.07,64342.61)	236.58 (-21.52,669.54)	98202.85 (-10258.7,264403.34)	318.02 (-33.02,856.88)	0.94 (0.93,0.96)	1518.8 (-134.22,4110.94)	16.19 (-1.42,43.88)	6950.28 (-705.02,18110.28)	22.75 (-2.29,59.32)	0.97 (0.89,1.05)	21242.31 (-1954.52,60171.56)	220.39 (-20.09,624.93)	91252.57 (-9548.46,246554.09)	295.28 (-30.71,798.40)	0.94 (0.93,0.96)
Central Sub-Saharan Africa	2340.26 (-167.59,6850.48)	91.73 (-6.54,270.01)	9272.47 (-685.06,27506.05)	159.33 (-11.59,472.47)	1.77 (1.73,1.81)	194.27 (-13.21,551.39)	8.24 (-0.55,23.30)	796.76 (-59.03,2277.22)	14.65 (-1.06,41.94)	1.84 (1.76,1.92)	2145.99 (-155,6287.45)	83.49 (-5.99,246.17)	8475.71 (-626.14,25137.48)	144.67 (-10.53,428.87)	1.76 (1.72,1.80)
East Asia	117571.63 (-9205.41,353937.85)	112.56 (-8.76,337.63)	609800.86 (-52711.32,1773473.64)	217.96 (-18.74,633.19)	2.41 (2.30,2.52)	4934.6 (-371.09,13749.9)	5.01 (-0.38,14.02)	31814.23 (-2636.75,86821.95)	11.58 (-0.95,31.69)	2.97 (2.85,3.10)	112637.03 (-8847,341004.09)	107.55 (-8.39,324.68)	577986.63 (-50097.86,1684827.18)	206.38 (-17.80,600.75)	2.38 (2.27,2.50)
Eastern Europe	77286.93 (-7137.61,214817.04)	213.77 (-19.43,595.78)	131348.95 (-13282.6,347540.11)	273.88 (-27.64,724.53)	0.87 (0.84,0.89)	9289.14 (-796.61,24729.03)	26.78 (-2.27,71.64)	17552.83 (-1672.72,45675.21)	37.01 (-3.52,96.37)	1.14 (1.11,1.18)	67997.8 (-6310.6,188348.04)	186.99 (-17.10,518.28)	113796.13 (-11564.84,303020.14)	236.87 (-24.03,630.59)	0.83 (0.80,0.85)
Eastern Sub-Saharan Africa	6676.78 (-498.17,20648.57)	78.83 (-5.84,243.30)	23957.01 (-1800.76,70837.5)	128.92 (-9.58,381.84)	1.62 (1.60,1.64)	569.54 (-40.94,1619.98)	7.18 (-0.51,20.38)	2172.82 (-162.17,6119.68)	12.32 (-0.91,34.81)	1.80 (1.76,1.84)	6107.23 (-458.84,18793.78)	71.65 (-5.34,219.89)	21784.18 (-1638.79,64189.82)	116.59 (-8.67,344.07)	1.60 (1.59,1.62)
High-income Asia Pacific	49789.78 (-3943,147417.36)	196.03 (-15.52,580.04)	147616.24 (-11325.75,434918.64)	251.23 (-19.58,739.18)	0.80 (0.79,0.82)	3491.73 (-272.5,9854.99)	13.79 (-1.08,38.93)	11482.55 (-889.21,32432.22)	19.32 (-1.52,54.55)	1.10 (1.01,1.19)	46298.06 (-3687.29,137837.97)	182.23 (-14.51,542.29)	136133.69 (-10457.66,400385.76)	231.91 (-18.10,681.10)	0.78 (0.76,0.80)
High-income North America	147950.97 (-14191.85,402285.47)	317.88 (-30.58,863.90)	345332.14 (-36899.36,912631.66)	390.27 (-41.82,1031.22)	0.38 (0.21,0.54)	22878.24 (-2260.82,63426.02)	49.06 (-4.86,135.96)	57189.5 (-6143.43,148017.9)	64.46 (-6.93,166.74)	1.01 (0.91,1.12)	125072.73 (-11900.93,341294.67)	268.82 (-25.66,733.54)	288142.64 (-30579.74,769221.86)	325.81 (-34.68,869.69)	0.25 (0.05,0.44)
North Africa and Middle East	35599.96 (-3229.55,99799.83)	184.41 (-16.41,518.06)	143897.05 (-15972.39,383797.71)	276.61 (-30.08,738.86)	1.31 (1.30,1.32)	2103.87 (-183.87,5624.46)	11.54 (-0.99,30.92)	9758.21 (-1037.13,25285.16)	19.60 (-2.03,50.85)	1.69 (1.65,1.72)	33496.09 (-3043.33,93912.3)	172.88 (-15.41,485.75)	134138.84 (-14924.31,357788.82)	257.01 (-28.03,686.17)	1.28 (1.27,1.29)
Oceania	513.47 (-47.59,1460.27)	153.81 (-13.88,436.77)	1607.54 (-152.44,4404.07)	195.32 (-18.15,538.53)	0.75 (0.70,0.80)	36.4 (-3.28,99.22)	11.78 (-1.03,32.24)	115.01 (-10.73,305.36)	14.91 (-1.36,39.67)	0.71 (0.63,0.78)	477.07 (-44.29,1367.56)	142.04 (-12.84,406.44)	1492.52 (-141.94,4106.75)	180.40 (-16.83,499.33)	0.75 (0.71,0.80)
South Asia	34265.96 (-2339.66,104274.01)	53.20 (-3.63,161.58)	204413.15 (-15004.78,593570.85)	114.02 (-8.32,332.27)	2.64 (2.59,2.69)	2087.71 (-138.17,6019.85)	3.36 (-0.22,9.69)	14255.48 (-989.83,40034.48)	8.15 (-0.57,22.94)	3.16 (3.06,3.27)	32178.25 (-2205.53,98110.1)	49.84 (-3.41,151.61)	190157.67 (-14009.86,553913.57)	105.87 (-7.76,309.49)	2.61 (2.56,2.66)
Southeast Asia	16491.65 (-1220.31,50234.32)	55.81 (-4.11,170.03)	89721.52 (-6878.79,266371.1)	111.66 (-8.43,330.80)	2.42 (2.37,2.48)	1257.41 (-87.38,3543.54)	4.45 (-0.31,12.58)	6975.79 (-507.74,19405.15)	9.03 (-0.65,25.24)	2.46 (2.41,2.51)	15234.24 (-1134.14,46602.86)	51.36 (-3.80,157.04)	82745.73 (-6413.03,244022.27)	102.63 (-7.83,302.24)	2.42 (2.36,2.48)
Southern Latin America	16583.88 (-1614.92,45463.93)	279.95 (-27.17,768.11)	41824.93 (-4497.83,108458.87)	371.36 (-40.02,962.63)	0.91 (0.86,0.97)	1869.23 (-177.83,4990.43)	31.73 (-3.01,84.88)	5466.65 (-586.26,14405.01)	48.44 (-5.20,127.63)	1.40 (1.30,1.51)	14714.64 (-1431.65,40429.79)	248.21 (-24.06,682.82)	36358.28 (-3905.55,95286.11)	322.91 (-34.77,845.76)	0.85 (0.80,0.89)
Southern Sub-Saharan Africa	6445.76 (-583.63,18038.47)	202.95 (-18.07,569.17)	19404.32 (-1911.46,52457.75)	284.13 (-27.67,769.55)	1.11 (1.09,1.13)	646.06 (-53.85,1748.18)	21.04 (-1.73,57.10)	2040.02 (-191.96,5239.08)	31.20 (-2.91,80.56)	1.37 (1.35,1.39)	5799.7 (-526.52,16124.2)	181.92 (-16.25,506.73)	17364.3 (-1710,47298.71)	252.93 (-24.64,689.58)	1.08 (1.06,1.11)
Tropical Latin America	23204.8 (-1983.69,64475.12)	214.97 (-18.18,599.85)	96978.46 (-9351.7,264993.38)	301.18 (-28.88,823.56)	1.13 (1.10,1.15)	1646.35 (-133.7,4445.61)	15.79 (-1.27,42.73)	7680.53 (-710.19,20474.23)	24.11 (-2.22,64.30)	1.44 (1.40,1.48)	21558.44 (-1848.46,59981.17)	199.18 (-16.90,555.46)	89297.92 (-8629.31,243719.04)	277.08 (-26.63,756.60)	1.10 (1.07,1.13)
Western Europe	200118.01 (-17889.52,565114.14)	260.84 (-23.43,735.80)	384254.77 (-36943.74,1042018.97)	320.43 (-31.17,868.01)	0.64 (0.60,0.69)	28390.89 (-2575.71,78098.12)	36.99 (-3.37,101.66)	59676.9 (-5821.54,159508.06)	49.30 (-4.86,131.32)	0.93 (0.85,1.01)	171727.13 (-15256.33,484640.77)	223.85 (-19.98,631.67)	324577.88 (-31055.85,884741.13)	271.13 (-26.24,738.54)	0.59 (0.55,0.63)
Western Sub-Saharan Africa	12750.57 (-983.56,38183.63)	125.33 (-9.56,374.93)	41746.02 (-3475.46,117890.09)	194.32 (-15.90,550.57)	1.39 (1.36,1.42)	1010.23 (-74.38,2823.52)	10.47 (-0.76,29.45)	3362.9 (-269.25,9112.56)	16.58 (-1.30,45.16)	1.36 (1.29,1.44)	11740.34 (-907.94,35038.67)	114.86 (-8.79,342.36)	38383.13 (-3204.34,108698.9)	177.74 (-14.58,504.64)	1.40 (1.37,1.42)

ASYR: Age-standardized Years lived with disability rate; YLDs: Years Lived with Disability; EAPC: Estimated Average Percentage Change; SDI: Socio-demographic Index.

Among the five distinct SDI regions, older adults in high-SDI areas consistently exhibited the highest ASYR for osteoarthritis and its two subtypes. However, the EAPC values in high-SDI regions were markedly lower than those in other SDI regions for both overall osteoarthritis and its subtypes. Over the past 32 years, the ASYR in all five SDI regions has demonstrated a consistently increasing trend (Figures 1A–C, 2B–D, Table 1).

### Regional trends in YLDs

**Figure 3:**
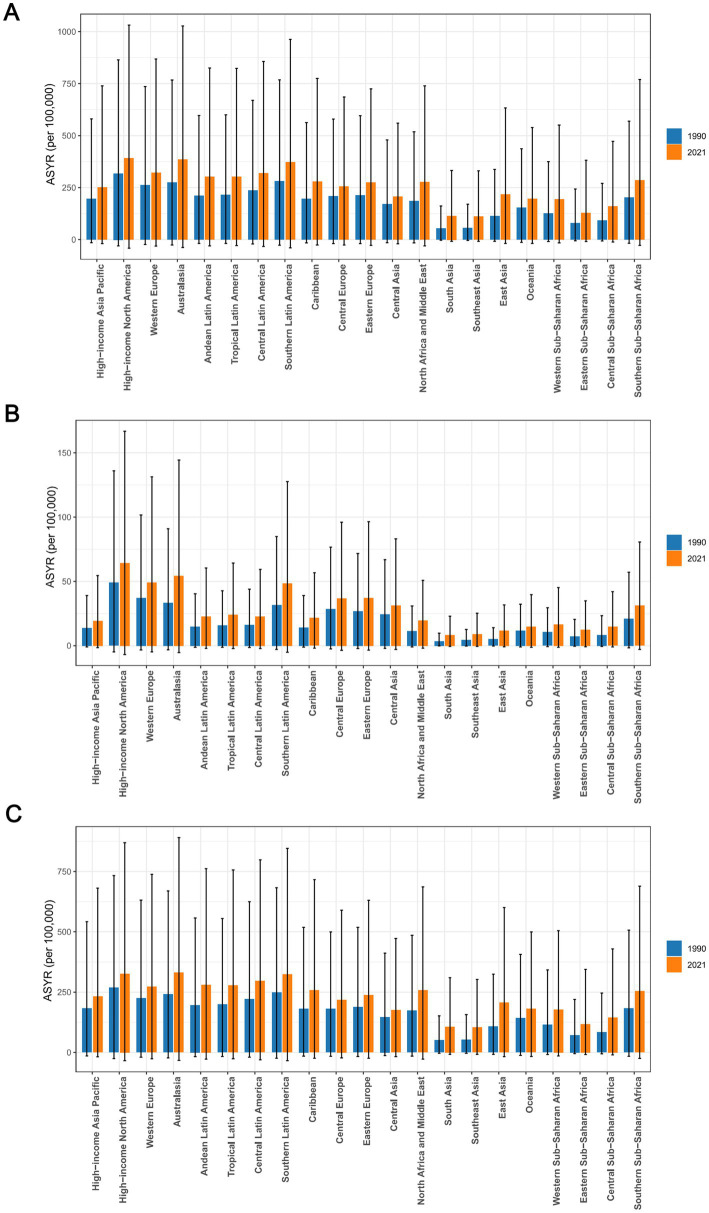
The ASYR of osteoarthritis **(A)**, hip osteoarthritis **(B)**, and knee osteoarthritis **(C)** among individuals aged 60 years and above across 21 regions in 1990 and 2021. ASYR, Age-standardized Years lived with disability rate.

In 2021, among the 21 regions, High-income North America recorded the highest ASYR of osteoarthritis, at 390.27 per 100,000 (95% UI: −41.82 to 1031.22). while Southeast Asia had the lowest ASYR, at 111.66 per 100,000 (95% UI: −8.43 to 330.80). Over the past 30 years, South Asia had the highest EAPC at 2.64 (95% CI: 2.59 to 2.69), ranking first among the 21 regions, while High-income North America had the lowest EAPC at 0.38 (95% CI: 0.21 to 0.54). Regarding osteoarthritis subtypes, in 2021, High-income North America had the highest ASYR for hip osteoarthritis, reaching 64.46 per 100,000 (95% CI: −6.93 to 166.74), while South Asia had the lowest ASYR for hip osteoarthritis at 8.15 per 100,000 (95% CI: −0.57 to 22.94). South Asia also had the highest EAPC for hip osteoarthritis at 2.64 (95% CI: 2.59 to 2.69), whereas Oceania had the lowest EAPC at 0.71 (95% CI: 0.63 to 0.78). For knee osteoarthritis, Australia recorded the highest ASYR in 2021, at 329.85 per 100,000 (95% UI: −32.74 to 890.47), while Southeast Asia had the lowest ASYR for knee osteoarthritis, at 102.63 per 100,000 (95% UI: −7.83 to 302.24). Once again, South Asia showed the highest EAPC for knee osteoarthritis at 2.61 (95% CI: 2.56 to 2.66), while High-income North America had the lowest EAPC at 0.25 (95% CI: 0.05 to 0.44) (Figures 1A–C, 3A–C, Table 1).

### National trends in YLDs

**Figure 4:**
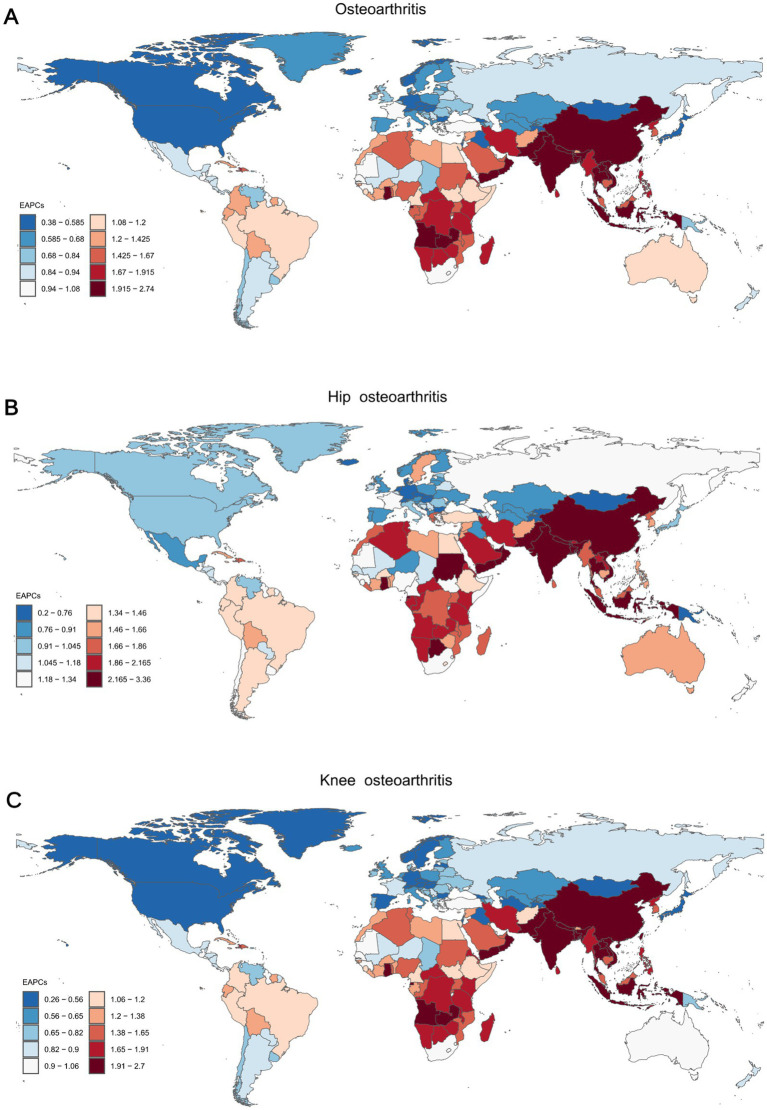
The EAPC of the ASYR for osteoarthritis **(A)**, hip osteoarthritis **(B)**, and knee osteoarthritis **(C)** among individuals aged 60 years and above across 204 countries and territories from 1990 to 2021. ASYR, Age-standardized Years lived with disability rate; EAPC, Estimated Average Percentage Changes.

Among 204 countries, China had the highest number of YLDs for osteoarthritis in older adults in 2021, with a total of 0.59 million (95% UI: −0.05 to 1.71). The United States recorded the highest YLDs for hip osteoarthritis at 5.32 ten thousand (95% UI: −0.58 to 13.71). For knee osteoarthritis, China led globally with 0.56 million YLDs (95% UI: −0.05 to 1.63). In terms of ASYR for overall osteoarthritis and its two subtypes, the United States had the highest rates globally, with an ASYR of 410.85 per 100,000 (95% UI: −44.47 to 1,083.52) for osteoarthritis, 67.23 per 100,000 (95% UI: −7.31 to 173.39) for hip osteoarthritis, and 343.62 per 100,000 (95% UI: −36.94 to 917.26) for knee osteoarthritis. On the other hand, the Democratic Republic of Timor-Leste had the lowest ASYR, with ASYRs for osteoarthritis, hip osteoarthritis, and knee osteoarthritis being 13.66 (−0.90 to 42.16), 0.90 (−0.05 to 2.58), and 12.76 (−0.85 to 39.37), respectively. Regarding EAPC, India showed the highest global rates for osteoarthritis and its two subtypes, with an EAPC of 2.74 (95% CI: 2.70 to 2.79) for osteoarthritis, 3.36 (95% CI: 3.25 to 3.48) for hip osteoarthritis, and 2.70 (95% CI: 2.65 to 2.75) for knee osteoarthritis. On the other hand, Georgia had the lowest EAPC for osteoarthritis, at 0.38 (95% CI: 0.38 to 0.39), Kingdom of Denmark had the lowest EAPC for hip osteoarthritis, at 0.21 (95% CI: −0.00 to 0.42), and the People’s Republic of Bangladesh had the lowest EAPC for knee osteoarthritis, at 3.07 (95% CI: 2.96 to 3.19). (Figures 4A–C, Supplementary Figures 1A–C, 2A–C, Supplementary Table 1).

### Net drift and local drift across different age groups

**Figure 5:**
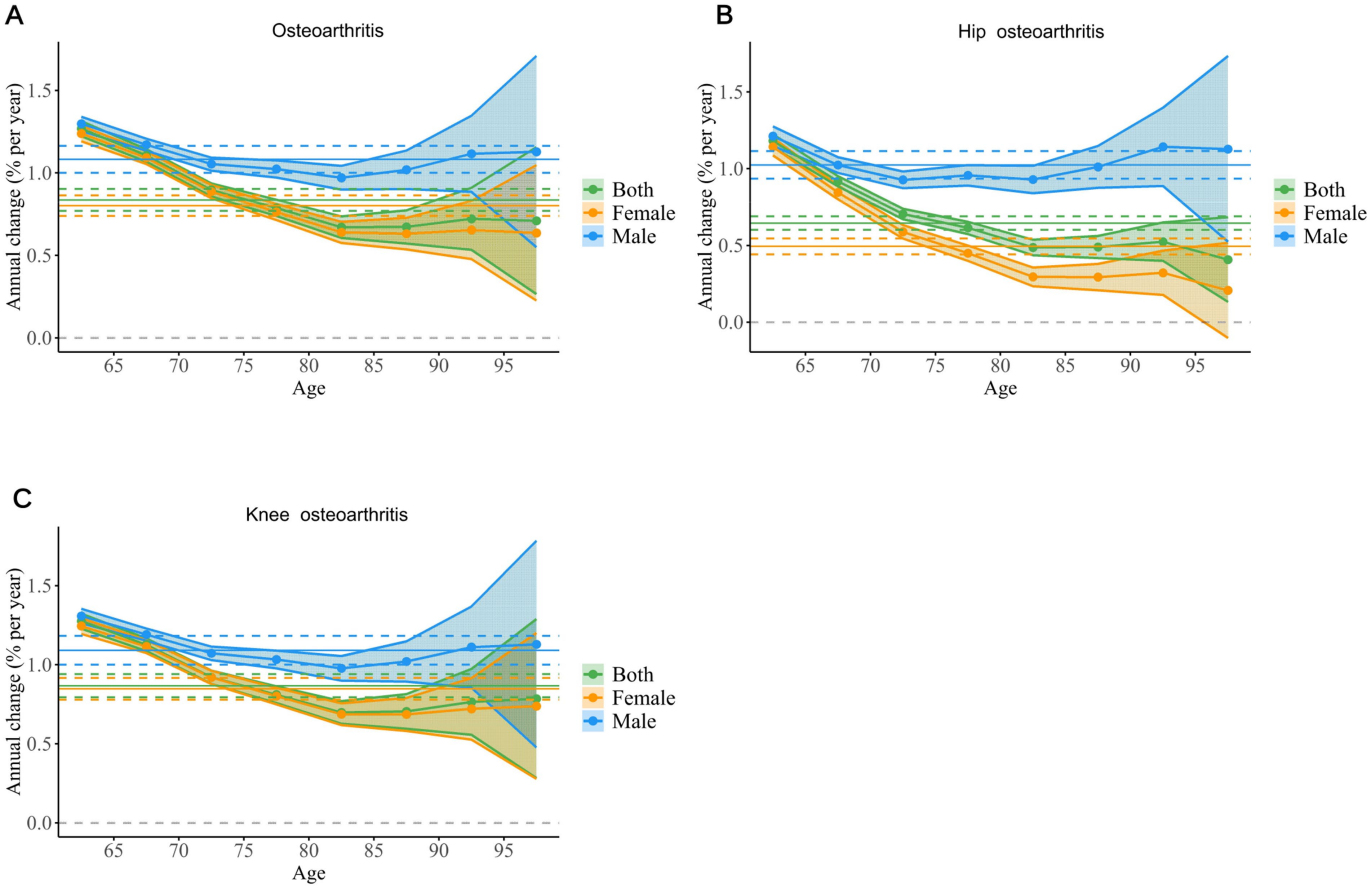
The local drift of YLD rates for osteoarthritis **(A)**, hip osteoarthritis **(B)**, and knee osteoarthritis **(C)** across eight age groups, stratified by sex, from 1992 to 2021. The dots and shaded areas denote the local drift (ie, annual percentage change of age-specific incidence, % per year) and their corresponding 95% CIs. YLD, Year Lived with Disability; CIs, Confidence Intervals.

The net drift for both males and females was positive, indicating an upward trend in the global ASYR for osteoarthritis among individuals aged 60 and above from 1990 to 2021. This suggests a progressively increasing burden of osteoarthritis in the older adult population worldwide. The local drift curves for both genders also showed an upward trend, signifying that the proportion of the osteoarthritis burden increased with age (Figure 5A).

From 1990 to 2021, the net drift for hip osteoarthritis in both males and females remained above zero, indicating a continuous rise in ASYR globally. Notably, the net drift value was higher in males, reflecting a faster rate of increase in the incidence of hip osteoarthritis among men. The local drift curve for males exhibited a U-shape, with significant increases in the 60–69 age group and after 85 years, suggesting an elevated burden of hip osteoarthritis in these age brackets. In contrast, the local drift for females showed a declining curve, indicating a slower progression of hip osteoarthritis and a reduced burden among women aged 60 and above (Figure 5B).

The global net drift for knee osteoarthritis was positive, meaning the ASYR for knee osteoarthritis in individuals aged 60 and above continued to rise. Similar to hip osteoarthritis, the rate of increase was faster in males. The local drift curves for both males and females demonstrated that the burden of knee osteoarthritis experienced a brief decline between the ages of 60 and 84, followed by stabilisation (Figure 5C).

### Impact of APC on osteoarthritis and its subtypes in older adults

**Figure 6:**
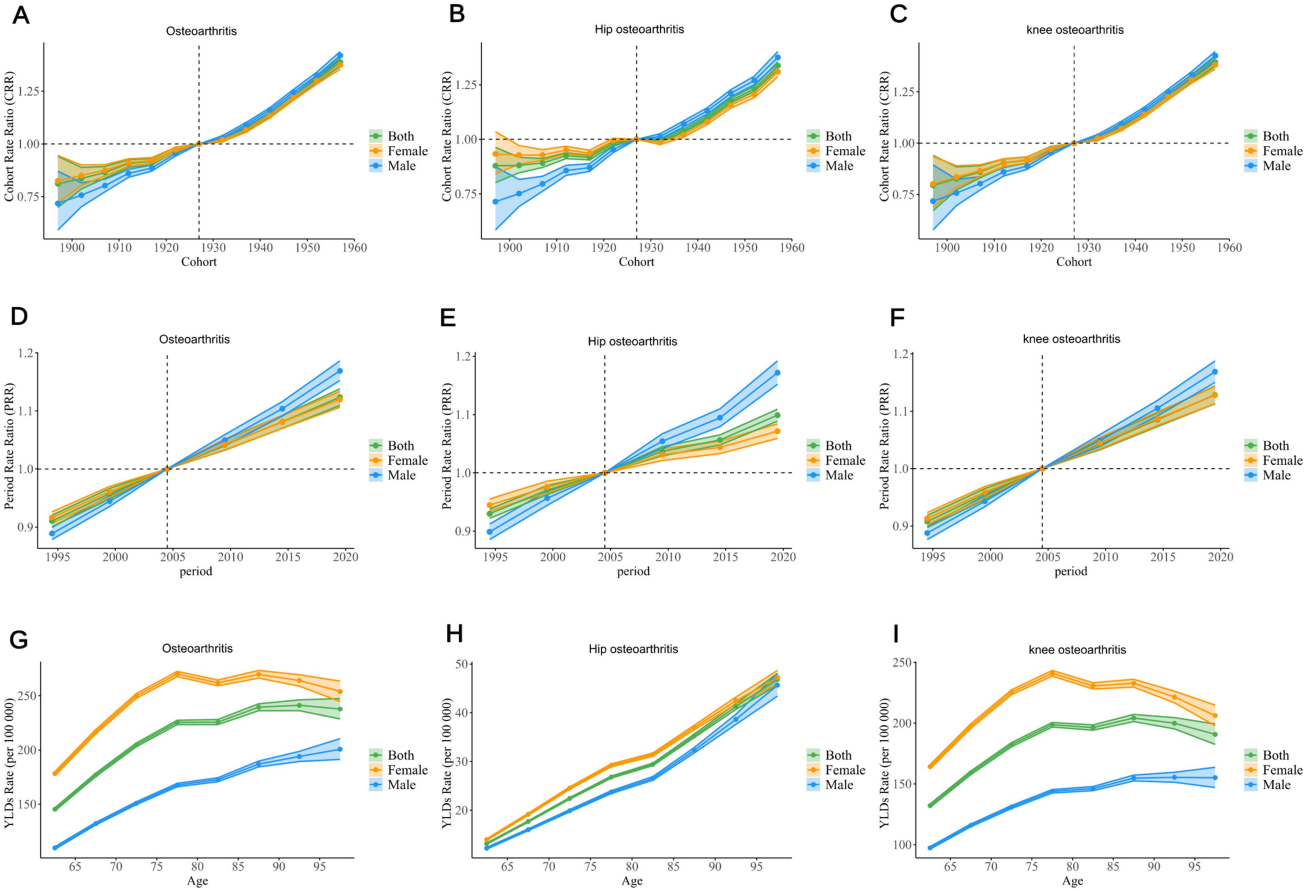
The APC model demonstrates the impact of age, period, and birth cohort on the YLD rates of osteoarthritis, hip arthritis, and knee arthritis among the older adult. The cohort effect is evidenced by the cohort-specific relative risk, with each cohort being compared to the reference cohort (1925–1929), while adjusting for the influence of age and period **(A–C)**. The PRR of each period group is compared with the reference period (2002–2006), with adjustments made for age and cohort effects **(D–F)**. The age effect is described through the longitudinal rate of a specific age, adjusted for variations across different birth cohorts and accounting for period-specific biases **(G–I)**. The dots and shaded areas denote the incidence rates or rate ratios and their corresponding 95% CIs. YLD, Year Lived with Disability; CIs, Confidence Intervals; PRR, Period Rate Ratios.

In terms of the CRR for osteoarthritis and its two subtypes, the CRR values for both males and females were close to 1, indicating that there was no significant difference in the incidence of osteoarthritis and its subtypes across different generations from 1895 to 1959. However, the global overall CRR, as well as that for males and females, showed an upward trend, reflecting an increasing burden of osteoarthritis in older adults, with men consistently having a higher risk than women (Figures 6A–C).

The PRR demonstrated a continuous increase in the relative risk of osteoarthritis and its two subtypes for the global population, males, and females since 1990, indicating a rising disease burden (Figures 6D–F).

The YLD rates for hip osteoarthritis in older adults showed a consistent upward trend, with a rapid increase in recent years. For osteoarthritis and knee osteoarthritis, the YLD rates for both genders and overall population exhibited an increasing trend in the 60–80 age group. In osteoarthritis, the YLD rates for females and the overall population remained stable after the age of 80, while males showed a gradual increase. For knee osteoarthritis, the YLD rates for females and the overall population showed a slight decline after the age of 80, in contrast to the previous trend, while males experienced a more stable and moderate increase (Figures 6G–I).

## Discussion

Osteoarthritis poses a significant global public health challenge, presenting particular risks to high-risk populations, including older adults, who may experience considerable joint pain, functional limitations, and a decline in quality of life. Osteoarthritis is a chronic degenerative disease that often leads to severe joint damage, affecting patients’ daily activities and increasing the risk of disability and healthcare burden among the older adult population. With the ageing population, the incidence of osteoarthritis continues to rise, particularly among individuals aged 60 and over, resulting in substantial economic and social burdens (7, 10, 15, 16). This vulnerability may also lead to a decline in self-care abilities, an increased risk of falls, and further health deterioration, making it a critical issue in global public health. Therefore, a comprehensive understanding of the epidemiological trends of osteoarthritis and its associated risk factors in older adults is essential for developing effective intervention strategies and achieving the World Health Organization’s 2030 Sustainable Development Goals. Accurate assessment of osteoarthritis trends within this population is crucial for disease evaluation and health target achievement. This study outlines the trends of osteoarthritis and its two subtypes using YLDs and ASYR, and explores their temporal dynamics using the APC model.

### Overall trends

Our findings indicate a clear upward trend in ASYR, period, and cohort for the two subtypes of osteoarthritis in older adults attributable to high BMI globally, from 1990 to 2021, with the greatest increase observed in knee osteoarthritis. Notably, the ASYR for hip osteoarthritis in older adults showed a sharp increase with age, whereas the ASYR for knee osteoarthritis exhibited a significant rise between the ages of 60 and 80, with no substantial increase observed in the population over 80 years. Specifically, the YLDs for knee osteoarthritis experienced the largest growth, increasing from 749,802.64 in 1990 to 2,295,374 in 2021, demonstrating that the knee joint is more susceptible to the negative effects of high BMI. As the largest weight-bearing joint in the body, the knee endures significant pressure, and high BMI increases the joint’s burden, leading to cartilage wear and a rising incidence of osteoarthritis. In contrast, while hip osteoarthritis also exhibited an upward trend, its increase was slightly lower than that of knee osteoarthritis, possibly due to the anatomical structure and functional characteristics of the hip joint. Previous guidelines and studies have shown that reducing BMI can effectively prevent the onset and progression of both knee and hip osteoarthritis (11, 17, 18). Research by Li JS et al. demonstrated that weight loss has been proven to significantly improve knee pain and functional impairment (19). A large longitudinal study by Gabby B. Joseph et al. also suggested that weight loss may prevent both radiographic and symptomatic knee osteoarthritis (20). Since 1980, the global average BMI in adults has continued to rise, and the problems of overweight and obesity have become increasingly severe (21).

### Differences across SDI regions

Among the five different SDI regions, older adults in high-SDI areas consistently had the highest ASYR for osteoarthritis and its two subtypes. This may be attributed to a higher degree of population ageing, better healthcare leading to higher diagnostic rates, and lifestyle factors in high-SDI regions. However, the EAPC in high-SDI regions was significantly lower than in other SDI regions, suggesting some success in controlling the rising burden of osteoarthritis. Possible reasons for this include more effective public health interventions, the promotion of healthier lifestyles, advances in early diagnosis, and improved treatment techniques. In contrast, the burden of osteoarthritis has been growing faster in low- and middle-SDI regions, with India having the highest global EAPC for osteoarthritis and its two subtypes. This rapid increase could be related to lifestyle changes brought on by fast economic development (e.g., the westernisation of diets and reduced physical activity), limited healthcare resources, and the accelerated pace of ageing in these countries. These findings indicate the need for enhanced osteoarthritis prevention and management in low-SDI countries, as well as the development of targeted public health strategies to address the rapidly growing disease burden.

### Regions-level trend analysis

In 2021, notable regional disparities in the burden of osteoarthritis were identified, with variations closely linked to socio-demographic factors, particularly the SDI and obesity trends. High-income regions such as North America and Western Europe, with higher SDI and rising obesity rates, exhibited a higher osteoarthritis burden, as reflected in the highest ASYR of osteoarthritis in High-income North America, reaching 390.27 per 100,000 (95%UI: −41.82 to 1031.22). These regions have experienced significant increases in obesity rates over the past three decades, which are strongly correlated with a rise in osteoarthritis cases, particularly in weight-bearing joints such as the knees and hips. In contrast, regions with lower SDI, such as South Asia and parts of Sub-Saharan Africa, have shown slower increases in obesity prevalence. Despite these regions having lower obesity rates, South Asia has demonstrated the highest EAPC of osteoarthritis at 2.64 (95%CI: 2.59 to 2.69) over the past 30 years, signaling a rapid increase in osteoarthritis cases. This is likely due to a combination of factors such as rapid urbanization, changes in lifestyle, and an aging population. Although obesity prevalence is lower in South Asia compared to high-income regions, other factors such as a growing urban population and improved healthcare access may contribute to the increase in osteoarthritis burden. The positive correlation between BMI and osteoarthritis remains evident, and as South Asia urbanizes and adopts more Westernized lifestyles, obesity rates are expected to rise, leading to further increases in osteoarthritis prevalence. The differences between regions highlight the complex interplay between socio-economic development, urbanization, and obesity trends, suggesting that a multifactorial approach is required to understand the burden of osteoarthritis. Regions with high SDI are experiencing a significant burden due to obesity and lifestyle factors, while regions with lower SDI, though starting with lower obesity rates, may see increases in osteoarthritis cases due to urbanization and demographic shifts.

### Country-level trend analysis

At the country level, China and India represent contrasting examples of osteoarthritis burden and trends, influenced by their SDI, obesity trends, and demographic factors. China ranks among the highest globally in terms of YLDs for osteoarthritis, reaching 0.59 million (95% UI: −0.05 to 1.71) in 2021, with knee osteoarthritis accounting for a substantial portion of this burden. China’s rapid urbanization and aging population, combined with a steady increase in obesity rates, are contributing to the rising prevalence of osteoarthritis. Although China’s obesity rates are not as high as those in high-income countries, they have been rising steadily over the past three decades, leading to an increase in osteoarthritis cases, particularly in older populations. This trend underscores the positive correlation between BMI and osteoarthritis, as higher obesity rates lead to more weight-bearing joint stress, contributing to higher osteoarthritis prevalence. The United States, with its high SDI and a high proportion of individuals with elevated BMI, also exhibits one of the highest osteoarthritis burdens globally, especially in the hip and knee osteoarthritis subtypes. The availability of extensive healthcare resources and diagnostic capabilities in the United States has led to higher diagnostic rates, further amplifying the observed osteoarthritis burden. The rising rates of obesity in the US have directly contributed to the increasing prevalence of osteoarthritis, reflecting the strong association between BMI and the disease. In contrast, India has the fastest-growing burden of osteoarthritis, as indicated by the highest EAPC at 2.64 (95%CI: 2.59 to 2.69). While India has a lower SDI and obesity rates compared to high-income countries, rapid urbanization, changing diets, and an aging population are driving the increase in osteoarthritis cases. The interaction between urbanization and rising obesity rates in India suggests that, as the population adopts more sedentary behaviors and less healthy diets, the prevalence of osteoarthritis is likely to continue to rise. Addressing obesity through public health interventions that promote active lifestyles and healthy diets will be crucial in managing the osteoarthritis burden in India. These country-level analyses highlight the importance of considering regional and national socio-economic factors in understanding the burden of osteoarthritis. Countries with higher SDI and obesity rates face an increasing burden due to the direct link between BMI and osteoarthritis, while countries like India, which are undergoing rapid socio-economic transitions, may experience a growing osteoarthritis burden as obesity rates rise alongside urbanization and demographic shifts.

### Differences in gender and age groups

This study found that the net drift for both males and females was positive, indicating a continuous increase in osteoarthritis among individuals aged 60 and above, with a faster rate of burden increase in males. The incidence of hip osteoarthritis in males showed particularly notable growth, especially in the 60–69 and over 85 age groups. This may be related to gender differences in physical activity patterns, workload, and physiological factors in these age brackets. For knee osteoarthritis, the YLD rates increased significantly between the ages of 60 and 80 but tended to stabilise or slightly decline after 80 years. This could be associated with changes in survival rates, health management in osteoarthritis patients, and the influence of other comorbidities in the older adult. Additionally, the burden of hip osteoarthritis in females aged 60 and above was significantly lower than in males, possibly due to differences in bone density, hormone levels, and higher health awareness and more proactive health behaviours among women.

### Cohort and period effects

Cohort and period effect analyses showed that the CRR for the global population, as well as for males and females, exhibited an upward trend, indicating an increasing incidence of osteoarthritis across different generations. This trend was particularly evident in populations with high BMI, likely due to the rising obesity rates, reduced physical activity, and unhealthy dietary patterns associated with modern lifestyles. Additionally, the continuous increase in PRR suggests that the relative risk of osteoarthritis and its subtypes has been growing globally, further emphasising its significance as a global public health issue.

### Limitations

Despite providing a comprehensive analysis of the global and regional trends in osteoarthritis burden, this study has several limitations. First, the reliance on data from the GBD study means that data quality and comparability may vary due to differences in national reporting systems. Data from some low-income countries may be incomplete, potentially introducing bias into the results. Second, the focus on high BMI as the sole risk factor limits the scope of the analysis, as other important risk factors, such as genetics, occupational exposure, and lifestyle, were not considered. This may underestimate the overall burden of osteoarthritis. Third, while YLDs reflect the impact of osteoarthritis on quality of life, they do not fully capture the broader socio-economic consequences of the disease, such as healthcare costs and productivity loss. Finally, the complexity of cohort and period effects poses challenges for interpretation. Although the APC model can reveal temporal dynamics, its results are influenced by multiple factors, introducing uncertainty and complexity to the analysis.

## Conclusion

Overall, this study reveals a significant increase in the burden of osteoarthritis among older adults attributable to high BMI over the past 32 years, both globally and across different SDI regions, particularly in countries such as China and India. Although high-SDI regions have a higher baseline burden, their slower growth rate indicates the effectiveness of existing management and prevention measures. The findings highlight the importance of controlling obesity, strengthening osteoarthritis prevention and management, and provide valuable insights for global public health policy development. Further research is needed to explore the multi-dimensional impact of osteoarthritis and optimise public health interventions to reduce the burden of this chronic disease among the global older adult population.

## Data Availability

Publicly available datasets were analyzed in this study. This data can be found here: data used for the analyses are publicly available from the Institute of Health Metrics and Evaluation (https://vizhub.healthdata.org/gbd-results/).

## Footnotes

^1^ http://ghdx.healthdata.org/gbd-results-tool

## Supplementary material

SUPPLEMENTARY FIGURE 1 In 2021, the ASYR of osteoarthritis **(A)**, hip osteoarthritis **(B)**, and knee osteoarthritis **(C)** among individuals aged 60 years and above across 204 countries and territories. ASYR, Age-standardized Years lived with disability rate.

SUPPLEMENTARY FIGURE 2 In 2021, the YLDs of osteoarthritis **(A)**, hip osteoarthritis **(B)**, and knee osteoarthritis **(C)** among individuals aged 60 years and above across 204 countries and territories. YLDs, Years Lived with Disability
