# Supplementary figures and images for: Global shifts in osteoarthritis subtype trends among older adults due to elevated BMI: an age-period-cohort analysis based on the global burden of disease database
Source: Front Public Health. 2025 Apr 28;13:1518572. doi: 10.3389/fpubh.2025.1518572 (PMC12066270; doi:10.3389/fpubh.2025.1518572)

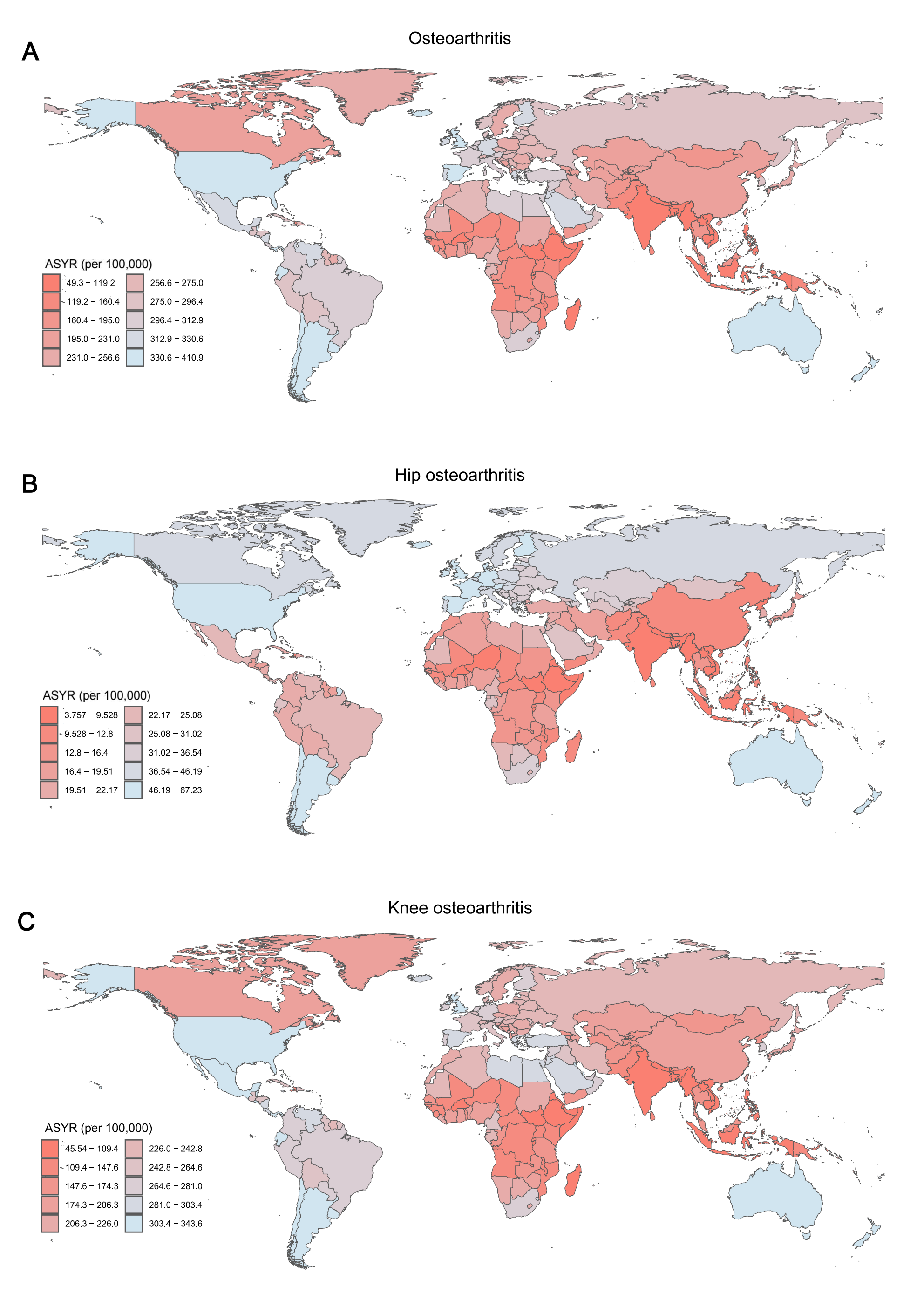

Supplement: SUPPLEMENTARY FIGURE 1 — In 2021, the ASYR of osteoarthritis (A), hip osteoarthritis (B), and knee osteoarthritis (C) among individuals aged 60 years and above across 204 countries and territories. ASYR, Age-standardized Years lived with disability rate. [file Image_1.tif]

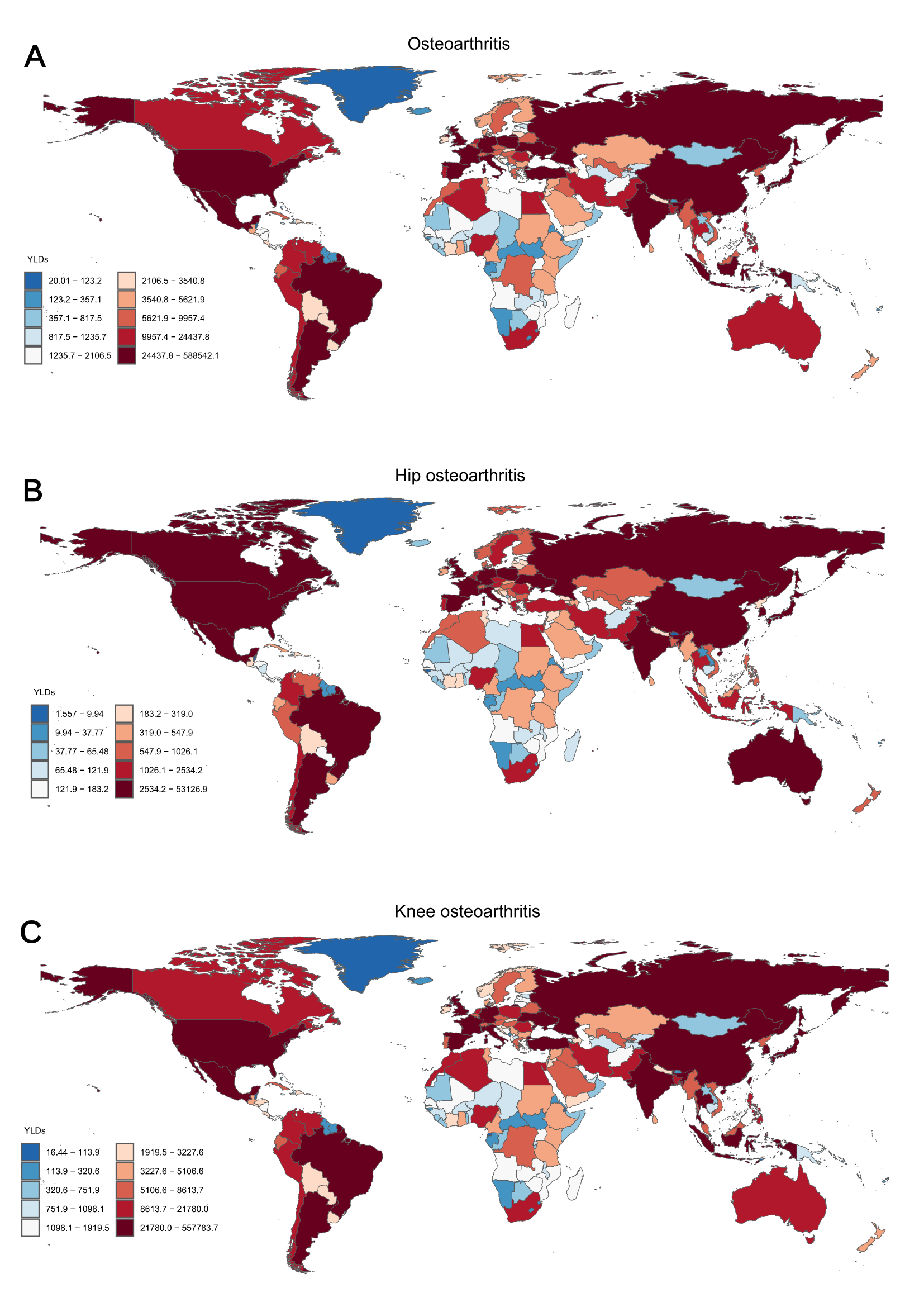

Supplement: SUPPLEMENTARY FIGURE 2 — In 2021, the YLDs of osteoarthritis (A), hip osteoarthritis (B), and knee osteoarthritis (C) among individuals aged 60 years and above across 204 countries and territories. YLDs, Years Lived with Disability [file Image_2.tiff]
