# Supplementary material for: Global shifts in osteoarthritis subtype trends among older adults due to elevated BMI: an age-period-cohort analysis based on the global burden of disease database
Source: Front Public Health. 2025 Apr 28;13:1518572. doi: 10.3389/fpubh.2025.1518572 (PMC12066270; doi:10.3389/fpubh.2025.1518572)
Supplement: Supplementary file 3 [file Data_Sheet_1.docx]

Supplement table 1. The YLDs and ASYR for 204 countries and territories in 1990 and 2021, along with the EAPC of ASYR from 1990 to 2021.

|  | Osteoarthritis | | | | | Osteoarthritis hip | | | | | Osteoarthritis knee | | | | |
| --- | --- | --- | --- | --- | --- | --- | --- | --- | --- | --- | --- | --- | --- | --- | --- |
|  | 1990 | | 2021 | | EAPC(95% CI) | 1990 | | 2021 | | EAPC(95% CI) | 1990 | | 2021 | | EAPC(95% CI) |
|  | No (95% UI) | ASR (per 100,000) | No (95% UI) | ASR (per 100,000) |  | No (95% UI) | ASR (per 100,000) | No (95% UI) | ASR (per 100,000) |  | No (95% UI) | ASR (per 100,000) | No (95% UI) | ASR (per 100,000) |  |
| American Samoa | 7.08(-0.74,18.86) | 279.34(-28.42,746.03) | 19.39(-2.18,50.02) | 338.55(-37.33,878.07) | 0.56(0.48,0.64) | 0.61(-0.06,1.61) | 25.46(-2.48,67.37) | 1.71(-0.18,4.4) | 31.03(-3.18,80.27) | 0.49(0.32,0.66) | 6.47(-0.68,17.42) | 253.88(-25.89,684.71) | 17.68(-2,45.99) | 307.52(-34.17,803.47) | 0.57(0.50,0.64) |
| Antigua and Barbuda | 15.03(-1.35,42.64) | 217.64(-20.06,617.36) | 39.27(-4.02,105.95) | 293.87(-29.71,795.2) | 0.99(0.97,1.00) | 1.07(-0.09,2.9) | 15.22(-1.32,41.38) | 2.87(-0.28,7.68) | 22.03(-2.15,58.98) | 1.21(1.19,1.23) | 13.96(-1.26,39.54) | 202.41(-18.72,573.10) | 36.4(-3.73,98.4) | 271.85(-27.50,736.42) | 0.97(0.96,0.98) |
| Arab Republic of Egypt | 6413.74(-596.92,17471.1) | 216.16(-19.47,591.67) | 22420.33(-2723.78,58299.93) | 311.67(-36.82,811.87) | 1.12(1.08,1.15) | 382.08(-34.47,1026.04) | 13.97(-1.22,37.66) | 1508.51(-172.71,3912.91) | 22.84(-2.54,58.98) | 1.46(1.39,1.54) | 6031.66(-562.02,16549.21) | 202.19(-18.24,556.55) | 20911.82(-2547.52,54605.94) | 288.83(-34.24,754.65) | 1.09(1.06,1.13) |
| Argentine Republic | 11571.2(-1128.78,31732.84) | 277.03(-26.95,760.69) | 26567.51(-2884.19,69422.89) | 367.3(-39.97,959.16) | 0.92(0.87,0.98) | 1289.54(-119.37,3427.02) | 31.06(-2.87,82.7) | 3428.3(-371.09,9075.05) | 47.3(-5.13,125.2) | 1.42(1.32,1.52) | 10281.66(-1002.94,28339.7) | 245.98(-23.92,679.08) | 23139.21(-2512.46,60819.58) | 320.00(-34.84,840.19) | 0.86(0.81,0.91) |
| Australia | 7095.11(-673.55,19940.89) | 273.66(-26.02,769.28) | 22919.85(-2266.53,61327.71) | 388.24(-38.54,1039.63) | 1.13(1.07,1.19) | 857.95(-82.97,2345.53) | 33.18(-3.21,90.7) | 3257.34(-327.31,8664.73) | 54.82(-5.53,145.79) | 1.66(1.55,1.76) | 6237.16(-589.36,17397.11) | 240.48(-22.76,670.94) | 19662.51(-1935.53,52949.36) | 333.42(-32.93,899.35) | 1.05(0.99,1.10) |
| Barbados | 98.62(-9.27,274.12) | 256.14(-24.71,707.19) | 233.33(-22.99,621.1) | 334.82(-32.89,891.7) | 0.85(0.81,0.89) | 7.4(-0.64,19.85) | 18.93(-1.68,50.83) | 18.06(-1.73,47.58) | 26.1(-2.49,68.78) | 1.03(0.98,1.07) | 91.23(-8.63,252.67) | 237.21(-23.04,653.14) | 215.26(-21.22,574.26) | 308.72(-30.35,823.88) | 0.83(0.79,0.87) |
| Belize | 29.24(-2.6,81.52) | 256.94(-22.8,716.49) | 115.68(-11.63,310.52) | 338.6(-33.9,910.29) | 0.88(0.79,0.96) | 2.17(-0.19,5.9) | 19.05(-1.67,51.85) | 9.17(-0.89,24.27) | 27.41(-2.65,72.74) | 1.15(1.00,1.31) | 27.08(-2.4,75.61) | 237.89(-21.11,664.49) | 106.51(-10.73,287.12) | 311.19(-31.22,839.88) | 0.85(0.77,0.93) |
| Bermuda | 22.88(-2.07,62.31) | 292.67(-26.34,798.32) | 67.57(-7.75,178.45) | 372.61(-43.12,982.98) | 0.78(0.75,0.81) | 1.81(-0.16,4.86) | 23.53(-2.02,63.18) | 5.61(-0.63,14.62) | 30.51(-3.45,79.38) | 0.82(0.78,0.86) | 21.07(-1.9,57.9) | 269.14(-24.21,740.54) | 61.95(-7.11,164.33) | 342.10(-39.62,906.38) | 0.78(0.75,0.80) |
| Bolivarian Republic of Venezuela | 2824.38(-276.89,7877) | 252.86(-24.48,707.4) | 12323.95(-1269.83,32551.29) | 325.16(-33.22,859.69) | 0.83(0.82,0.84) | 181.66(-16.01,488.38) | 16.62(-1.45,44.68) | 830.83(-80.14,2180.06) | 22.28(-2.13,58.5) | 0.97(0.95,0.99) | 2642.72(-260.05,7373.74) | 236.24(-22.97,661.01) | 11493.13(-1185.14,30290.06) | 302.88(-30.98,798.86) | 0.82(0.81,0.83) |
| Bosnia and Herzegovina | 861.23(-79.6,2419.88) | 180.41(-15.99,508.05) | 1954.66(-205.27,5336.16) | 230.76(-24.11,630.03) | 0.80(0.74,0.85) | 102.72(-9.02,284.07) | 22.73(-1.92,62.86) | 262.46(-25.56,704.51) | 31.34(-3.03,84.11) | 1.08(0.99,1.16) | 758.51(-70.57,2133.34) | 157.68(-14.07,444.27) | 1692.2(-177.69,4617.8) | 199.41(-20.84,544.31) | 0.75(0.70,0.81) |
| Brunei Darussalam | 18.75(-1.45,54.64) | 171.91(-13.38,501.47) | 122.99(-11.47,347.4) | 294.2(-26.6,832.82) | 1.82(1.73,1.90) | 1.33(-0.1,3.78) | 12.28(-0.91,34.98) | 9.43(-0.84,26.52) | 22.76(-1.96,64.18) | 2.17(2.06,2.29) | 17.41(-1.36,50.81) | 159.63(-12.48,466.16) | 113.57(-10.58,320.58) | 271.44(-24.51,768.43) | 1.79(1.70,1.87) |
| Burkina Faso | 333.57(-27.88,999.05) | 64.3(-5.3,192.12) | 991.29(-75.61,2969.77) | 93.72(-7.1,280.73) | 1.23(1.19,1.27) | 25.19(-1.87,73.95) | 5.13(-0.37,15) | 80.28(-5.57,226.44) | 7.92(-0.55,22.34) | 1.46(1.43,1.50) | 308.38(-25.93,934.98) | 59.17(-4.91,179.12) | 911.02(-70.3,2749.43) | 85.80(-6.57,258.82) | 1.21(1.17,1.25) |
| Canada | 7244.52(-704.73,19859.46) | 170.03(-16.53,466.12) | 21437.77(-2078.58,57205.26) | 221.82(-21.53,591.73) | 0.58(0.42,0.74) | 1199.72(-114.36,3282.15) | 28.17(-2.69,77.18) | 4058.08(-389.83,10890.52) | 41.81(-4.03,112.15) | 0.92(0.73,1.11) | 6044.8(-592.33,16690.97) | 141.85(-13.90,391.80) | 17379.69(-1671.04,47074.17) | 180.01(-17.31,487.65) | 0.51(0.35,0.66) |
| Central African Republic | 90.13(-6.52,274.62) | 70.8(-5.05,215.16) | 279.23(-21.82,811.95) | 121.43(-8.96,358.25) | 1.79(1.75,1.83) | 7.04(-0.47,21.22) | 5.98(-0.39,18.09) | 22.12(-1.54,62.77) | 10.44(-0.7,30.23) | 1.88(1.84,1.93) | 83.1(-6.04,252.74) | 64.81(-4.64,196.98) | 257.12(-20.3,752.35) | 110.99(-8.26,328.09) | 1.78(1.74,1.82) |
| Commonwealth of Dominica | 21.69(-2.09,59.54) | 278.41(-26.92,763.23) | 37.21(-4.05,97.32) | 347.11(-37.52,908.69) | 0.68(0.62,0.74) | 1.52(-0.14,4.07) | 19.67(-1.78,52.67) | 2.8(-0.29,7.15) | 26.63(-2.77,68.05) | 0.98(0.93,1.02) | 20.17(-1.95,55.4) | 258.74(-25.09,709.70) | 34.4(-3.74,90.59) | 320.48(-34.68,844.47) | 0.66(0.60,0.72) |
| Commonwealth of the Bahamas | 44.67(-4.07,124.67) | 253.36(-22.97,708.42) | 161.83(-16.87,433.61) | 330.62(-34.05,888.08) | 0.85(0.82,0.88) | 3.29(-0.28,9.03) | 18.92(-1.6,51.98) | 12.1(-1.17,31.79) | 25.42(-2.44,66.88) | 0.98(0.94,1.03) | 41.38(-3.8,116.02) | 234.45(-21.43,658.38) | 149.72(-15.67,403.89) | 305.20(-31.54,824.37) | 0.84(0.81,0.87) |
| Cook Islands | 3.92(-0.42,10.5) | 263.71(-27.45,709.78) | 11.65(-1.33,30.46) | 341.78(-38.68,895.33) | 0.83(0.77,0.89) | 0.34(-0.03,0.9) | 23.51(-2.23,62.43) | 1.05(-0.11,2.73) | 31.14(-3.22,80.78) | 0.81(0.70,0.92) | 3.58(-0.38,9.63) | 240.20(-25.13,648.60) | 10.6(-1.21,27.77) | 310.64(-35.36,815.54) | 0.83(0.78,0.88) |
| Czech Republic | 4139.89(-426.16,11209.58) | 225.69(-23.12,612.1) | 7503.06(-832.05,19979.46) | 261.08(-29.25,694.78) | 0.47(0.47,0.48) | 549.2(-53.37,1467.36) | 30.62(-2.96,81.79) | 1091.93(-116.46,2863.19) | 37.45(-4.05,98.31) | 0.67(0.65,0.68) | 3590.69(-371.5,9762.88) | 195.07(-20.11,530.63) | 6411.13(-710.83,17158) | 223.63(-25.05,598.33) | 0.44(0.43,0.45) |
| Democratic People's Republic of Korea | 1687.99(-141.82,5006.29) | 97.02(-8.08,286.68) | 6770.32(-594.34,20221.05) | 173.55(-15.16,517.65) | 1.89(1.79,2.00) | 74.55(-5.75,217.24) | 4.54(-0.35,13.16) | 295.54(-22.99,836.42) | 7.75(-0.6,21.93) | 1.74(1.62,1.86) | 1613.44(-136.12,4811.13) | 92.48(-7.73,274.82) | 6474.77(-571.41,19311.88) | 165.80(-14.57,493.82) | 1.90(1.80,2.00) |
| Democratic Republic of Sao Tome and Principe | 12.71(-1.11,36.01) | 156.36(-13.51,443.23) | 27.97(-2.8,78.09) | 228.51(-22.28,637.96) | 1.25(1.22,1.29) | 0.98(-0.08,2.79) | 12.44(-1.01,35.56) | 2.33(-0.2,6.36) | 19.97(-1.72,54.65) | 1.56(1.52,1.60) | 11.74(-1.03,33.42) | 143.92(-12.50,410.15) | 25.64(-2.6,71.73) | 208.54(-20.52,582.98) | 1.22(1.19,1.26) |
| Democratic Republic of the Congo | 1634.58(-114.06,4766.97) | 91.28(-6.31,268.67) | 6205.11(-455.23,18493.61) | 160.13(-11.62,476.36) | 1.80(1.75,1.85) | 136.43(-9.28,386.72) | 8.26(-0.55,23.46) | 526.87(-38.47,1527.85) | 14.55(-1.05,42.14) | 1.78(1.68,1.89) | 1498.15(-105.38,4367.39) | 83.02(-5.79,244.43) | 5678.24(-416.79,16877.12) | 145.58(-10.57,431.35) | 1.80(1.76,1.85) |
| Democratic Republic of Timor-Leste | 8.32(-0.7,25.61) | 30.27(-2.58,93.12) | 53.87(-3.59,165.04) | 49.3(-3.3,150.89) | 1.72(1.62,1.82) | 0.62(-0.05,1.87) | 2.42(-0.19,7.32) | 3.96(-0.26,11.61) | 3.76(-0.25,10.99) | 1.55(1.45,1.66) | 7.7(-0.65,23.59) | 27.85(-2.38,85.41) | 49.91(-3.33,152.8) | 45.54(-3.06,139.41) | 1.73(1.63,1.83) |
| Democratic Socialist Republic of Sri Lanka | 968.77(-68.27,2878.75) | 76.5(-5.36,226.34) | 4678.76(-343.45,13643.81) | 130.38(-9.43,379.77) | 1.76(1.72,1.81) | 73.67(-4.83,213.76) | 6.06(-0.4,17.62) | 358.03(-24.65,1026.48) | 10.24(-0.69,29.44) | 1.74(1.72,1.77) | 895.1(-63.39,2668.06) | 70.45(-4.96,208.98) | 4320.73(-318.21,12588.2) | 120.14(-8.72,349.75) | 1.76(1.71,1.81) |
| Dominican Republic | 742.87(-57.46,2117.22) | 169.93(-13.12,485.25) | 3198.05(-296.8,9016.86) | 263.97(-24.44,744.68) | 1.47(1.43,1.52) | 51.59(-3.9,145.65) | 12.11(-0.91,34.3) | 241.26(-20.76,659.37) | 19.99(-1.72,54.65) | 1.74(1.69,1.79) | 691.28(-53.92,1954.69) | 157.81(-12.28,447.46) | 2956.79(-275.25,8344.82) | 243.98(-22.66,689.06) | 1.45(1.41,1.50) |
| Eastern Republic of Uruguay | 1409.31(-132.62,3869.17) | 271.08(-25.54,744.07) | 2516.82(-249.98,6711.54) | 352.3(-35.34,937.98) | 0.84(0.79,0.89) | 159.52(-14.63,437.17) | 30.74(-2.82,84.24) | 332.85(-32.02,882.2) | 46.21(-4.5,122.25) | 1.33(1.24,1.42) | 1249.79(-116.78,3464.31) | 240.34(-22.48,666.17) | 2183.97(-217.59,5862.22) | 306.09(-30.81,820.44) | 0.77(0.73,0.82) |
| Federal Democratic Republic of Ethiopia | 1456.67(-114.47,4303.21) | 65.07(-5.11,192.88) | 4530.12(-335.29,13162.6) | 93.48(-6.89,272.02) | 1.20(1.15,1.24) | 125.17(-8.97,368.44) | 6.05(-0.43,17.77) | 431.99(-31.14,1236.27) | 9.25(-0.67,26.58) | 1.43(1.37,1.49) | 1331.5(-105.32,3962.46) | 59.02(-4.65,176.14) | 4098.13(-303.72,12039.61) | 84.23(-6.21,247.68) | 1.17(1.13,1.22) |
| Federal Democratic Republic of Nepal | 506.67(-34.38,1497.97) | 45.88(-3.12,136.01) | 2744.8(-197.49,7915.9) | 93.28(-6.66,270.1) | 2.48(2.43,2.54) | 28.89(-1.74,84.3) | 2.68(-0.16,7.82) | 187.41(-12.84,550.93) | 6.48(-0.44,19.05) | 3.09(3.02,3.16) | 477.78(-32.63,1418.04) | 43.20(-2.95,128.57) | 2557.4(-184.7,7402.55) | 86.80(-6.22,252.38) | 2.44(2.39,2.49) |
| Federal Republic of Germany | 46781.62(-4260.96,130059.32) | 283.97(-25.85,787.28) | 80950.42(-7676.85,216626.6) | 323.94(-31.15,864.19) | 0.40(0.38,0.42) | 6577.35(-579.69,18117.44) | 39.87(-3.51,109.93) | 12679.01(-1177.36,33478.81) | 50.3(-4.73,132.27) | 0.70(0.65,0.75) | 40204.27(-3634.17,112113.75) | 244.10(-22.04,679.50) | 68271.41(-6481.96,184593.66) | 273.65(-26.34,737.82) | 0.35(0.33,0.36) |
| Federal Republic of Nigeria | 6886.52(-536.13,20472.55) | 133.71(-10.32,396.92) | 20986.78(-1822.02,58425.75) | 214.42(-18.24,598.52) | 1.50(1.44,1.56) | 564.56(-41.13,1576.23) | 11.51(-0.83,32.28) | 1670.87(-136.43,4556.96) | 18.08(-1.45,49.41) | 1.25(1.11,1.39) | 6321.95(-494.18,18788.11) | 122.20(-9.47,361.98) | 19315.91(-1679.3,53860.9) | 196.33(-16.73,548.91) | 1.53(1.47,1.59) |
| Federal Republic of Somalia | 162.11(-11.9,491.83) | 71.36(-5.25,216.97) | 682.78(-51.07,1945.8) | 103.59(-7.73,296.09) | 1.20(1.16,1.23) | 12.99(-0.94,37.53) | 6.16(-0.44,17.79) | 53.73(-3.57,153.47) | 8.89(-0.61,25.37) | 1.20(1.16,1.24) | 149.12(-11,455.75) | 65.20(-4.82,199.47) | 629.05(-47.48,1783.29) | 94.71(-7.11,269.99) | 1.20(1.16,1.23) |
| Federated States of Micronesia | 13.56(-1.48,36.71) | 224.01(-23.88,609.67) | 26.46(-3.07,68.77) | 295.41(-32.38,780.42) | 0.85(0.77,0.93) | 1.05(-0.11,2.81) | 17.89(-1.75,48.01) | 1.95(-0.21,5.14) | 23.46(-2.4,61.97) | 0.75(0.62,0.89) | 12.51(-1.37,33.91) | 206.12(-22.12,561.45) | 24.51(-2.85,63.8) | 271.95(-29.92,718.03) | 0.86(0.78,0.93) |
| Federative Republic of Brazil | 22602.15(-1927.68,62741.8) | 214.63(-18.11,598.35) | 94864.55(-9120.13,259238.1) | 301.24(-28.8,823.78) | 1.13(1.10,1.16) | 1604.55(-130.11,4335.76) | 15.78(-1.27,42.74) | 7525.13(-693.96,20067.55) | 24.15(-2.21,64.43) | 1.45(1.41,1.49) | 20997.6(-1796.21,58381.82) | 198.85(-16.83,554.16) | 87339.42(-8413.95,238424.62) | 277.09(-26.55,756.80) | 1.10(1.07,1.13) |
| French Republic | 24376.44(-2085.6,71309.23) | 223.42(-19.23,653.52) | 54582.95(-4957.41,151883.23) | 301.22(-27.56,836.78) | 0.96(0.87,1.04) | 3603.41(-318.17,10135.76) | 32.96(-2.92,92.58) | 8869.76(-811.33,23967.68) | 48.44(-4.44,130.75) | 1.24(0.97,1.50) | 20773.03(-1762.4,60499.03) | 190.46(-16.26,555.09) | 45713.2(-4132.3,126624.79) | 252.78(-23.02,700.18) | 0.90(0.85,0.96) |
| Gabonese Republic | 116.61(-10.28,325.73) | 165.57(-14.53,463.54) | 311.02(-32.26,846.75) | 260.99(-26.44,714.21) | 1.43(1.38,1.48) | 10.28(-0.84,27.89) | 15.19(-1.23,41.36) | 31.14(-3.09,84.37) | 27.52(-2.66,74.68) | 1.90(1.84,1.96) | 106.33(-9.42,297.3) | 150.37(-13.26,421.26) | 279.88(-29.11,755.86) | 233.47(-23.73,632.59) | 1.38(1.33,1.43) |
| Georgia | 1408.83(-121.07,3956.15) | 176.85(-15.15,499.07) | 1607.07(-151.94,4463.89) | 197.69(-18.68,549) | 0.38(0.37,0.39) | 203.99(-17.06,569.55) | 26.37(-2.19,73.68) | 247.29(-21.3,664.86) | 29.96(-2.59,80.45) | 0.45(0.43,0.47) | 1204.84(-103.46,3374.03) | 150.49(-12.89,423.65) | 1359.78(-129.31,3795.82) | 167.73(-15.93,468.30) | 0.36(0.35,0.37) |
| Grand Duchy of Luxembourg | 186.51(-15.64,528.25) | 260.25(-21.87,736.93) | 427.94(-40.78,1166.59) | 320.21(-30.74,872.09) | 0.65(0.63,0.68) | 26.33(-2.14,72.2) | 36.8(-3,101.06) | 66.53(-6.44,178.22) | 49.55(-4.83,132.63) | 0.94(0.89,0.98) | 160.18(-13.52,453.65) | 223.45(-18.90,632.59) | 361.41(-34.3,986.8) | 270.66(-25.87,738.22) | 0.60(0.58,0.63) |
| Greenland | 6.66(-0.65,18.2) | 191.37(-18.17,524.33) | 20.01(-1.92,53.41) | 230.97(-21.9,621.3) | 0.60(0.56,0.64) | 1.05(-0.1,2.83) | 30.85(-2.95,83) | 3.57(-0.32,9.44) | 41.95(-3.76,111.08) | 1.02(0.94,1.09) | 5.61(-0.54,15.38) | 160.52(-15.15,439.82) | 16.44(-1.57,44.04) | 189.03(-17.92,509.08) | 0.51(0.49,0.54) |
| Grenada | 18.21(-1.59,52.81) | 195.06(-17.28,565.29) | 38.34(-3.79,105.62) | 275.01(-26.87,760) | 1.13(1.08,1.18) | 1.22(-0.1,3.34) | 12.83(-1.1,35.04) | 2.7(-0.25,7.22) | 19.83(-1.8,53.28) | 1.43(1.38,1.47) | 16.99(-1.49,49.38) | 182.23(-16.19,529.22) | 35.64(-3.53,97.56) | 255.18(-25.02,700.82) | 1.11(1.06,1.16) |
| Guam | 21.05(-2.13,57.59) | 238.03(-23.64,654.98) | 82.87(-8.95,224.36) | 300.12(-32.15,812.77) | 0.76(0.72,0.79) | 1.72(-0.17,4.55) | 20.73(-1.97,55.21) | 7.18(-0.72,18.81) | 25.97(-2.59,68.04) | 0.75(0.69,0.80) | 19.34(-1.95,53.26) | 217.30(-21.54,601.54) | 75.69(-8.2,206.49) | 274.16(-29.48,748.03) | 0.76(0.73,0.79) |
| Hashemite Kingdom of Jordan | 325.12(-31.46,889.77) | 234.46(-22.41,642.1) | 2583.25(-323.54,6751.33) | 321.9(-39.23,840.84) | 1.05(1.03,1.07) | 19.83(-1.75,52.51) | 15.1(-1.33,40.18) | 181.06(-22.62,465.7) | 23.81(-2.87,61.3) | 1.49(1.45,1.54) | 305.29(-29.67,837.07) | 219.36(-21.05,601.53) | 2402.19(-300.71,6329.32) | 298.08(-36.34,784.54) | 1.02(1.00,1.04) |
| Hellenic Republic | 5030.38(-468.5,14015.33) | 250.24(-23.35,696.86) | 9957.43(-997.71,27107.2) | 328.44(-32.85,892.74) | 0.95(0.88,1.02) | 657.3(-59.86,1808.12) | 32.78(-2.98,90.17) | 1420.78(-143.6,3772.62) | 46.17(-4.68,122.33) | 1.84(1.52,2.15) | 4373.08(-406.86,12212.43) | 217.46(-20.27,607.01) | 8536.65(-850.31,23412.41) | 282.27(-28.07,773.70) | 0.83(0.77,0.88) |
| Hungary | 4411.12(-398.79,12199.55) | 224.76(-20.18,623.88) | 7099.59(-727.98,19029.81) | 267.81(-27.97,717.73) | 0.58(0.56,0.59) | 589.93(-49.61,1577.73) | 30.98(-2.6,83.2) | 1026.15(-95.54,2684.79) | 38.16(-3.61,99.9) | 0.69(0.67,0.71) | 3821.19(-349.6,10625.32) | 193.78(-17.61,540.49) | 6073.44(-628.59,16467.21) | 229.64(-24.20,621.86) | 0.56(0.55,0.57) |
| Independent State of Papua New Guinea | 250.19(-22.36,730.54) | 117.09(-10.32,342.07) | 833.51(-70.11,2339.53) | 149.69(-12.49,424.28) | 0.76(0.72,0.80) | 16.28(-1.39,46.31) | 8.25(-0.69,23.62) | 55.12(-4.52,150.36) | 10.54(-0.86,28.98) | 0.72(0.66,0.78) | 233.92(-20.95,687.79) | 108.84(-9.62,320.25) | 778.39(-65.66,2186.02) | 139.16(-11.64,394.76) | 0.76(0.73,0.80) |
| Independent State of Samoa | 26.7(-3,69.24) | 255.88(-28.12,667.86) | 54(-6.35,139.25) | 314(-36.2,814.72) | 0.61(0.56,0.67) | 2.14(-0.22,5.63) | 21.3(-2.12,56.26) | 4.31(-0.47,11.14) | 25.9(-2.77,67.26) | 0.52(0.42,0.62) | 24.55(-2.78,63.86) | 234.59(-26.00,613.47) | 49.7(-5.87,128.38) | 288.10(-33.41,749.08) | 0.62(0.57,0.67) |
| Ireland | 1359.29(-122.89,3811.8) | 249.31(-22.84,699.26) | 3303.22(-324.46,8921.9) | 321.8(-31.94,869.26) | 0.83(0.79,0.87) | 192.69(-16.87,527.44) | 35.4(-3.13,96.82) | 514.73(-49.63,1376.28) | 49.97(-4.86,133.52) | 1.10(1.04,1.16) | 1166.61(-105.96,3333.16) | 213.91(-19.68,611.29) | 2788.49(-273.66,7541.31) | 271.82(-26.95,735.50) | 0.78(0.74,0.81) |
| Islamic Republic of Afghanistan | 1065.86(-86.46,3040.27) | 124.06(-9.86,353.47) | 1430.41(-118.04,4094.91) | 175.31(-14.61,499.38) | 1.22(1.14,1.30) | 55.31(-4.25,156.59) | 6.85(-0.51,19.27) | 84.72(-6.69,237.57) | 10.63(-0.84,29.89) | 1.59(1.47,1.72) | 1010.55(-82.26,2885.68) | 117.21(-9.34,334.22) | 1345.69(-111.16,3852.58) | 164.68(-13.75,469.53) | 1.20(1.12,1.27) |
| Islamic Republic of Iran | 4676.8(-386.1,13264.85) | 151.22(-12.27,429.92) | 23303.72(-2433.54,62556.26) | 255.66(-26.29,687.04) | 1.69(1.67,1.71) | 287.36(-22.76,787.16) | 9.97(-0.78,27.3) | 1664.93(-161.88,4355.41) | 18.84(-1.8,49.43) | 2.00(1.92,2.08) | 4389.44(-363.03,12494.2) | 141.25(-11.48,402.68) | 21638.79(-2270.75,57961.52) | 236.82(-24.48,635.06) | 1.67(1.64,1.69) |
| Islamic Republic of Mauritania | 213.47(-18.34,613.09) | 184.01(-15.85,527.55) | 649.05(-60.33,1793.62) | 258.08(-23.71,714.19) | 1.05(1.02,1.09) | 17.59(-1.38,49.44) | 15.85(-1.23,44.6) | 58.58(-5.23,160.85) | 24.35(-2.16,66.55) | 1.34(1.29,1.39) | 195.88(-16.85,559.9) | 168.16(-14.50,480.10) | 590.47(-54.97,1635.29) | 233.72(-21.51,647.28) | 1.03(1.00,1.06) |
| Islamic Republic of Pakistan | 4956.5(-397.93,14643.9) | 74.95(-6.01,221.61) | 19139.11(-1531.94,56231.71) | 141.99(-11.26,417.92) | 2.38(2.25,2.51) | 383.76(-30.24,1094.01) | 5.91(-0.47,16.83) | 1645.81(-123.33,4510.8) | 12.52(-0.93,34.47) | 2.75(2.63,2.87) | 4572.74(-368.74,13655.81) | 69.03(-5.56,206.38) | 17493.3(-1408.63,51651.09) | 129.47(-10.33,383.02) | 2.35(2.21,2.48) |
| Jamaica | 504.83(-47.73,1428.81) | 218.96(-20.85,619.69) | 1188.87(-115.53,3181.54) | 306.07(-29.66,819.25) | 1.15(1.11,1.20) | 35.23(-3,98.03) | 15.17(-1.3,42.26) | 90.76(-8.31,243.69) | 22.99(-2.11,61.67) | 1.44(1.36,1.51) | 469.6(-44.44,1321.45) | 203.78(-19.41,573.53) | 1098.11(-107.02,2943.77) | 283.08(-27.51,758.84) | 1.13(1.09,1.17) |
| Japan | 42936.32(-3354.67,128054.58) | 196.81(-15.39,586.77) | 107916.08(-8290.96,320294.69) | 237.21(-18.55,702.7) | 0.56(0.53,0.58) | 3037.09(-235.79,8580.66) | 13.96(-1.08,39.42) | 8572.58(-653.7,24227.45) | 18.61(-1.44,52.47) | 0.93(0.82,1.04) | 39899.22(-3131.23,119411.11) | 182.85(-14.36,547.19) | 99343.49(-7654.67,293212.85) | 218.61(-17.16,643.91) | 0.53(0.50,0.55) |
| Kingdom of Bahrain | 37.49(-3.73,103.81) | 212.21(-20.22,591.74) | 292.69(-34.81,759.48) | 304.66(-34.89,794.37) | 1.18(1.17,1.19) | 2.33(-0.21,6.19) | 14.25(-1.24,38.11) | 20.47(-2.33,52.84) | 23.23(-2.52,60.48) | 1.57(1.54,1.59) | 35.16(-3.52,97.57) | 197.97(-18.98,552.62) | 272.22(-32.47,705.22) | 281.43(-32.32,733.31) | 1.15(1.14,1.16) |
| Kingdom of Belgium | 4803.18(-408.38,13528.38) | 234.61(-19.96,660.98) | 9097.74(-855.11,24929.33) | 301.72(-28.6,825.24) | 0.78(0.74,0.81) | 666.1(-58.49,1853.1) | 32.54(-2.86,90.49) | 1384.09(-134.26,3719.71) | 45.48(-4.46,122) | 1.05(0.99,1.11) | 4137.08(-348.67,11721.23) | 202.06(-17.05,573.19) | 7713.65(-721.31,21202.42) | 256.23(-24.16,702.89) | 0.73(0.70,0.76) |
| Kingdom of Bhutan | 33.07(-3.07,96) | 120.8(-10.98,352.44) | 132.66(-11.86,373.26) | 181.51(-16.17,511.24) | 1.42(1.38,1.46) | 2.02(-0.16,5.74) | 7.59(-0.61,21.57) | 9.76(-0.84,26.57) | 13.46(-1.16,36.68) | 2.01(1.95,2.07) | 31.06(-2.91,90.28) | 113.21(-10.34,330.86) | 122.9(-11,345.3) | 168.05(-14.99,472.80) | 1.38(1.34,1.41) |
| Kingdom of Cambodia | 249.6(-18.77,757.03) | 47.55(-3.58,144.18) | 1155.57(-91.36,3493.21) | 76.25(-5.93,230.91) | 1.62(1.57,1.66) | 17.09(-1.24,51.01) | 3.42(-0.25,10.22) | 78.18(-5.73,225.35) | 5.4(-0.39,15.65) | 1.56(1.50,1.62) | 232.52(-17.55,709.43) | 44.13(-3.33,134.49) | 1077.39(-85.47,3267.52) | 70.85(-5.53,215.13) | 1.62(1.57,1.67) |
| Kingdom of Denmark | 2536.03(-220.05,7140.16) | 236.81(-20.83,664.39) | 4490.18(-406.16,12315.85) | 287.71(-26.34,789.23) | 0.62(0.57,0.67) | 478.87(-41.09,1336.34) | 44.69(-3.87,124.81) | 757.28(-64.69,2055.82) | 48.25(-4.15,130.31) | 0.20(-0.01,0.40) | 2057.16(-178.68,5882.83) | 192.13(-16.92,546.91) | 3732.91(-339.82,10362.5) | 239.46(-22.06,664.32) | 0.71(0.68,0.75) |
| Kingdom of Eswatini | 68.45(-6.81,189.9) | 225.52(-22.21,625.86) | 193.94(-19.93,517.31) | 317.14(-32.51,844.01) | 1.06(0.97,1.15) | 6.51(-0.57,17.48) | 22.63(-1.99,60.72) | 19.63(-1.93,51.62) | 34.25(-3.36,89.57) | 1.24(1.09,1.39) | 61.94(-6.23,172.48) | 202.88(-20.20,564.58) | 174.31(-17.97,464.21) | 282.89(-29.09,751.20) | 1.04(0.96,1.12) |
| Kingdom of Lesotho | 175.2(-16.55,477.97) | 174.12(-16.21,474.41) | 307.79(-30.29,828.72) | 241.22(-23.24,651.24) | 1.02(0.97,1.07) | 14.61(-1.24,40.82) | 15.06(-1.26,42.16) | 27.75(-2.55,73.73) | 23.07(-2.06,61.57) | 1.39(1.35,1.43) | 160.6(-15.3,437.68) | 159.06(-14.94,433.27) | 280.04(-27.77,757.36) | 218.15(-21.19,591.53) | 0.99(0.93,1.04) |
| Kingdom of Morocco | 2659.96(-263.41,7586.85) | 156.81(-15.31,447.51) | 9754.04(-1011.32,26521.6) | 232.47(-23.71,634.94) | 1.30(1.29,1.31) | 152.31(-13.67,413.85) | 9.31(-0.82,25.35) | 632.64(-59.95,1654.05) | 15.73(-1.46,41.3) | 1.72(1.70,1.74) | 2507.66(-249.1,7138.95) | 147.50(-14.45,419.99) | 9121.41(-949.6,24918.34) | 216.74(-22.21,594.57) | 1.27(1.26,1.28) |
| Kingdom of Norway | 2124.49(-177.91,5965.41) | 231.83(-19.57,650.69) | 3638.08(-333.26,10155.95) | 279.93(-25.72,779.68) | 0.57(0.55,0.59) | 303.39(-25.4,823.87) | 32.98(-2.77,89.37) | 563.69(-51.55,1543.4) | 43.11(-3.95,118.05) | 0.86(0.85,0.88) | 1821.1(-152.14,5119.9) | 198.85(-16.76,558.98) | 3074.39(-280.31,8619.36) | 236.82(-21.66,662.89) | 0.52(0.50,0.55) |
| Kingdom of Saudi Arabia | 1242.21(-123.74,3449.11) | 202.49(-20.06,564.35) | 5613.89(-665.19,14496.07) | 322.12(-37.03,833.42) | 1.48(1.41,1.55) | 78.46(-7.49,212.26) | 13.35(-1.24,36.21) | 407.94(-46.29,1044.52) | 25.31(-2.77,64.75) | 2.07(2.00,2.14) | 1163.75(-115.99,3243.56) | 189.14(-18.77,528.35) | 5205.95(-619.3,13520.41) | 296.81(-34.27,771.28) | 1.43(1.36,1.50) |
| Kingdom of Spain | 20597.92(-2033.1,57517.89) | 280.39(-27.77,782.9) | 43340.8(-4492.76,115405.54) | 351.42(-37.06,935.84) | 0.59(0.51,0.68) | 2988.08(-293.06,8155.52) | 40.76(-4,111.17) | 6934.34(-724.79,18252.72) | 55.66(-5.95,146) | 0.85(0.69,1.01) | 17609.84(-1732.55,48396.68) | 239.64(-23.66,658.91) | 36406.46(-3750.16,97981.11) | 295.76(-30.97,796.02) | 0.55(0.48,0.62) |
| Kingdom of Sweden | 3990.08(-347.66,11199.34) | 198.37(-17.52,558.56) | 6965.35(-655.82,19458.39) | 250.53(-24.05,701.75) | 0.67(0.59,0.75) | 522.88(-46.02,1420.85) | 25.85(-2.3,70.34) | 1225.08(-112.09,3342.13) | 43.78(-4.05,118.91) | 1.53(1.04,2.03) | 3467.2(-301.31,9847.91) | 172.52(-15.20,492.07) | 5740.26(-537.99,16063.39) | 206.75(-19.72,579.47) | 0.48(0.34,0.62) |
| Kingdom of Thailand | 3094.86(-252.7,9206.7) | 76.33(-6.13,226.25) | 23746.63(-1979.43,67755) | 166.44(-13.82,475.49) | 2.71(2.63,2.78) | 219.25(-16.87,619.34) | 5.64(-0.43,15.96) | 1825.41(-143.04,5061.3) | 12.93(-1.01,35.86) | 2.87(2.81,2.93) | 2875.6(-235.86,8574.47) | 70.69(-5.70,209.87) | 21921.21(-1847.28,62237.91) | 153.51(-12.89,436.56) | 2.69(2.61,2.77) |
| Kingdom of the Netherlands | 6814.31(-645.7,18909.68) | 260.52(-24.79,722.39) | 14587.98(-1427.32,39695.74) | 312.88(-30.95,851.57) | 0.58(0.56,0.60) | 1002.37(-93.6,2763.92) | 38.26(-3.59,105.41) | 2189.59(-213.15,5890.15) | 46.72(-4.58,125.67) | 0.62(0.52,0.72) | 5811.95(-551.52,16215.62) | 222.25(-21.18,620.10) | 12398.4(-1212.6,33713.57) | 266.17(-26.34,724.66) | 0.58(0.56,0.59) |
| Kingdom of Tonga | 17.49(-1.85,47.13) | 258.12(-26.84,699.23) | 32.42(-3.78,84.93) | 336.08(-39.01,881.36) | 0.79(0.70,0.88) | 1.38(-0.13,3.6) | 21.15(-2.02,55.43) | 2.65(-0.28,6.88) | 27.71(-2.95,71.92) | 0.69(0.52,0.87) | 16.11(-1.72,43.54) | 236.97(-24.82,643.69) | 29.77(-3.49,78.16) | 308.37(-36.04,810.43) | 0.80(0.72,0.88) |
| Kyrgyz Republic | 612.61(-56.44,1710.75) | 168.74(-15.29,472.84) | 1121.42(-113.59,2983.97) | 203.73(-20.08,545.65) | 0.60(0.58,0.61) | 82.52(-6.79,225.02) | 23.42(-1.89,63.96) | 155.44(-14.63,413.22) | 29.64(-2.71,79.31) | 0.71(0.68,0.74) | 530.09(-49.6,1469.55) | 145.32(-13.38,404.83) | 965.98(-98.63,2576.3) | 174.09(-17.31,466.44) | 0.58(0.56,0.59) |
| Lao People's Democratic Republic | 127.62(-9.43,379.44) | 53.1(-3.87,157.73) | 494.53(-36.69,1443.85) | 93.14(-6.89,272.04) | 2.01(1.95,2.07) | 9.28(-0.63,27.49) | 4.07(-0.27,12.01) | 37.04(-2.69,108.23) | 7.3(-0.53,21.33) | 2.09(2.04,2.15) | 118.34(-8.81,351.92) | 49.03(-3.60,145.75) | 457.49(-34,1329.35) | 85.84(-6.36,249.56) | 2.00(1.94,2.06) |
| Lebanese Republic | 533.67(-50.31,1466.96) | 204.96(-19.21,564.57) | 2140.65(-233.21,5548.96) | 288.91(-32.05,747.92) | 1.09(1.00,1.19) | 32.67(-2.91,86.85) | 13.14(-1.17,35.1) | 158.81(-16.92,411.75) | 20.89(-2.27,54.18) | 1.41(1.14,1.68) | 501(-47.34,1380.48) | 191.82(-18.01,528.79) | 1981.84(-216.19,5147.26) | 268.02(-29.76,695.72) | 1.07(0.98,1.16) |
| Malaysia | 1113.61(-88.36,3246.69) | 104.47(-8.24,305.1) | 6054.88(-556.39,17019.45) | 170.31(-15.29,480.28) | 1.61(1.55,1.67) | 88.61(-6.3,251.44) | 8.52(-0.61,24.19) | 506.66(-43.41,1379.03) | 14.75(-1.23,40.32) | 1.84(1.79,1.90) | 1025(-82.29,2999.03) | 95.96(-7.65,281.22) | 5548.22(-512.98,15572.07) | 155.56(-14.06,438.06) | 1.59(1.53,1.65) |
| Mongolia | 175.84(-15.59,504.91) | 145.89(-12.72,419.52) | 418.2(-40.25,1151.32) | 174.3(-16.2,482.27) | 0.47(0.40,0.54) | 23.94(-1.99,65.8) | 20.65(-1.69,56.87) | 57.64(-5.22,155.5) | 25.5(-2.24,69.26) | 0.70(0.68,0.72) | 151.89(-13.44,438.5) | 125.24(-10.92,362.14) | 360.56(-34.74,992.32) | 148.80(-13.87,411.03) | 0.43(0.35,0.51) |
| Montenegro | 172.79(-16.97,473.28) | 226.88(-22.37,623.03) | 357.07(-37.79,945.51) | 269.69(-28.56,713.7) | 0.60(0.59,0.61) | 23.59(-2.16,62.35) | 31.62(-2.88,83.88) | 48.68(-4.75,128.32) | 38.24(-3.72,100.74) | 0.71(0.67,0.74) | 149.2(-14.76,414.52) | 195.26(-19.42,543.55) | 308.39(-32.74,817.85) | 231.45(-24.59,613.48) | 0.58(0.57,0.60) |
| New Zealand | 1439(-139.04,3938.59) | 276.46(-26.7,757.29) | 4020.25(-430.78,10733.35) | 361.7(-38.87,966.04) | 0.90(0.87,0.93) | 177.33(-16.37,478.81) | 34.14(-3.15,92.17) | 565.45(-59.83,1489.49) | 50.69(-5.38,133.45) | 1.34(1.28,1.41) | 1261.67(-121.38,3474.04) | 242.33(-23.31,667.52) | 3454.8(-368.87,9334.35) | 311.01(-33.30,841.13) | 0.84(0.81,0.86) |
| North Macedonia | 457.91(-43.33,1247.39) | 202.22(-19.01,553.6) | 1073.25(-107.48,2932.5) | 243.88(-24.15,666.42) | 0.64(0.63,0.66) | 59.38(-5.34,159.56) | 27.21(-2.43,73.23) | 140.48(-13.54,365.81) | 33.95(-3.21,88.52) | 0.77(0.76,0.79) | 398.53(-37.73,1093.02) | 175.01(-16.46,481.28) | 932.78(-93.88,2563) | 209.93(-20.92,576.57) | 0.62(0.61,0.63) |
| Northern Mariana Islands | 3.76(-0.42,10.24) | 251.8(-26.7,690.29) | 19.79(-2.33,51.36) | 320.77(-36.01,841.43) | 0.75(0.67,0.83) | 0.33(-0.03,0.85) | 23.21(-2.27,61.01) | 1.65(-0.19,4.32) | 28.33(-3.1,74.37) | 0.51(0.38,0.64) | 3.44(-0.38,9.36) | 228.59(-24.26,628.07) | 18.15(-2.14,47.13) | 292.44(-32.91,766.64) | 0.77(0.70,0.85) |
| Palestine | 225.09(-23.66,608.45) | 218.17(-22.57,591.16) | 780.25(-93.8,2039.06) | 283.48(-33.18,742.28) | 0.82(0.80,0.83) | 13.39(-1.42,35.7) | 13.53(-1.39,36.06) | 49.81(-5.92,129.05) | 19.14(-2.17,49.67) | 1.05(1.01,1.09) | 211.7(-22.21,574.58) | 204.65(-21.14,556.54) | 730.44(-87.74,1905.97) | 264.35(-30.94,691.08) | 0.80(0.79,0.82) |
| People's Democratic Republic of Algeria | 2436.8(-211.54,7065.41) | 162.38(-13.96,472.09) | 10899.83(-1130.78,29400.83) | 257.57(-26.36,695.14) | 1.51(1.49,1.53) | 137.66(-11.54,375.54) | 9.83(-0.82,27) | 734.13(-71.66,1929.07) | 18.19(-1.75,47.89) | 2.04(2.01,2.06) | 2299.13(-200.04,6662.44) | 152.55(-13.14,442.95) | 10165.7(-1054.02,27342.93) | 239.39(-24.50,644.35) | 1.47(1.45,1.49) |
| People's Republic of Bangladesh | 2162.38(-168.64,6551.82) | 40.48(-3.13,122.53) | 15055.22(-1125.33,44943.62) | 88(-6.56,263.13) | 2.71(2.63,2.79) | 125.82(-9.11,367.42) | 2.4(-0.17,7) | 1009.47(-69.8,2947.15) | 6(-0.42,17.48) | 3.23(3.14,3.33) | 2036.56(-159.77,6228.48) | 38.08(-2.96,116.32) | 14045.75(-1055.59,41908.07) | 82.00(-6.15,245.08) | 2.68(2.59,2.76) |
| People's Republic of China | 112918.52(-8832.83,339799.08) | 112.14(-8.72,336.3) | 588542.12(-50835.76,1712477.21) | 217.84(-18.71,633.13) | 2.43(2.31,2.55) | 4702.8(-353.46,13074.86) | 4.95(-0.37,13.83) | 30758.39(-2546.45,83907.23) | 11.59(-0.95,31.72) | 3.03(2.89,3.16) | 108215.71(-8490.34,327169.98) | 107.19(-8.35,323.23) | 557783.73(-48311.79,1627000.88) | 206.25(-17.77,600.71) | 2.40(2.28,2.52) |
| Plurinational State of Bolivia | 691.95(-58.51,1965.09) | 184.41(-15.46,526.23) | 3099.7(-298.47,8492.19) | 277.3(-26.32,761.43) | 1.33(1.28,1.38) | 44.39(-3.63,123.47) | 12.23(-0.99,34.06) | 215.27(-18.66,576.47) | 19.88(-1.71,53.46) | 1.61(1.56,1.67) | 647.55(-54.87,1845.53) | 172.18(-14.46,492.92) | 2884.43(-277.96,7936.34) | 257.42(-24.46,709.63) | 1.31(1.26,1.35) |
| Portuguese Republic | 4460.2(-405.18,12648.09) | 237.33(-21.56,673.57) | 10101.55(-1019.64,27080.95) | 315.79(-32.3,845.96) | 0.84(0.77,0.90) | 594.21(-53.92,1662.96) | 31.76(-2.88,89.01) | 1487.85(-150.89,3998.51) | 46.02(-4.71,123.79) | 0.91(0.77,1.06) | 3865.99(-351.16,10962.53) | 205.57(-18.68,583.37) | 8613.7(-865.49,23380.72) | 269.77(-27.46,731.82) | 0.82(0.76,0.88) |
| Principality of Andorra | 18.31(-1.75,51.07) | 247.25(-23.49,689.72) | 58.51(-5.37,159.69) | 303.57(-27.9,828.19) | 0.69(0.68,0.71) | 2.65(-0.24,7.16) | 36.02(-3.28,97.29) | 9.21(-0.86,24.75) | 47.59(-4.47,127.85) | 0.94(0.89,0.99) | 15.66(-1.5,44.05) | 211.23(-20.09,593.86) | 49.3(-4.51,135.22) | 255.98(-23.43,702.09) | 0.65(0.63,0.66) |
| Principality of Monaco | 28.1(-2.54,77.54) | 297.75(-27.14,821.8) | 44.59(-4.36,119.35) | 348.93(-34.4,931.36) | 0.50(0.48,0.52) | 4.22(-0.39,11.42) | 44.43(-4.17,119.95) | 7.18(-0.71,19.23) | 55.65(-5.55,149) | 0.71(0.66,0.75) | 23.88(-2.14,65.87) | 253.32(-22.90,699.43) | 37.41(-3.65,100.53) | 293.27(-28.86,785.67) | 0.46(0.45,0.47) |
| Puerto Rico | 1375.59(-129.1,3770.5) | 294.51(-27.64,806.73) | 3540.8(-371.67,9495.33) | 374.07(-40,1003.17) | 0.81(0.76,0.86) | 109.96(-10.46,293.86) | 23.73(-2.25,63.43) | 313.2(-32.9,817.15) | 31.78(-3.41,82.83) | 1.03(0.95,1.10) | 1265.62(-118.66,3463.06) | 270.78(-25.40,740.41) | 3227.59(-338.58,8680.12) | 342.29(-36.57,919.48) | 0.79(0.74,0.84) |
| Republic of Albania | 463.57(-45.47,1280.91) | 195.14(-19,539.35) | 1414.14(-146.24,3793.78) | 237.37(-24.47,636.89) | 0.66(0.64,0.67) | 58.01(-5.39,156.72) | 25.4(-2.34,68.9) | 189.34(-18.36,495.19) | 32.63(-3.15,85.57) | 0.88(0.86,0.90) | 405.57(-40.16,1113.73) | 169.73(-16.69,465.06) | 1224.8(-127.01,3289.49) | 204.74(-21.18,549.90) | 0.62(0.61,0.63) |
| Republic of Angola | 331.33(-25.14,994.64) | 78.81(-5.91,236.94) | 1820.65(-130.63,5310.91) | 143.69(-10.1,421.23) | 1.94(1.88,1.99) | 26.57(-1.82,76.69) | 6.82(-0.47,19.69) | 156.11(-11.64,446.53) | 13.2(-0.95,37.83) | 2.16(2.09,2.24) | 304.76(-23.32,909.05) | 71.99(-5.43,215.54) | 1664.54(-119.23,4844.8) | 130.49(-9.16,382.82) | 1.92(1.86,1.97) |
| Republic of Armenia | 560.84(-52.46,1531.24) | 173.73(-16.06,477.03) | 1251.83(-128.6,3375.61) | 210.96(-21.49,567.55) | 0.66(0.65,0.68) | 75.56(-7.02,205.81) | 24.37(-2.23,66.85) | 185.67(-17.43,497.24) | 31.87(-2.98,85.22) | 0.93(0.90,0.95) | 485.28(-45.44,1336.18) | 149.35(-13.83,413.07) | 1066.15(-110.77,2873.56) | 179.10(-18.44,481.53) | 0.62(0.60,0.63) |
| Republic of Austria | 4109.85(-345.12,11345.16) | 260.41(-21.93,719.44) | 7195.6(-664.77,19848.32) | 310.83(-29.31,858.25) | 0.54(0.53,0.55) | 576.44(-47.54,1593.11) | 36.51(-3,100.64) | 1103.48(-100.97,2957.62) | 47.26(-4.41,126.5) | 0.82(0.80,0.85) | 3533.4(-299.69,9801.76) | 223.90(-19.08,620.79) | 6092.12(-559.22,16884.6) | 263.57(-24.67,731.22) | 0.49(0.48,0.50) |
| Republic of Azerbaijan | 956.97(-80.04,2688.94) | 170(-14.01,478.21) | 2592.52(-256.11,6899.57) | 214.35(-20.41,576.15) | 0.80(0.77,0.82) | 131.79(-10.91,353.24) | 24.16(-1.95,65.01) | 377.98(-35.5,1006.87) | 33.11(-3.02,88.52) | 1.10(1.06,1.15) | 825.18(-69.24,2316.8) | 145.84(-12.07,410.41) | 2214.54(-219.38,5901.32) | 181.24(-17.32,487.52) | 0.74(0.72,0.76) |
| Republic of Belarus | 3540.73(-331.65,9727.41) | 210.83(-19.68,578.55) | 5921.89(-649.53,15633.4) | 271.59(-29.7,717.33) | 0.85(0.84,0.87) | 417.35(-36.74,1123.69) | 25.48(-2.24,68.71) | 753.44(-74.04,1972.22) | 34.87(-3.4,91.2) | 1.07(1.05,1.10) | 3123.38(-293.61,8585.57) | 185.35(-17.37,508.95) | 5168.45(-571.07,13624.24) | 236.72(-26.09,624.22) | 0.82(0.81,0.84) |
| Republic of Benin | 303.93(-24.95,890.55) | 127.84(-10.39,374.73) | 1098.47(-86.36,3228.59) | 191.63(-14.83,563.99) | 1.28(1.26,1.30) | 22.67(-1.78,63.5) | 9.8(-0.76,27.51) | 90.77(-6.76,255.05) | 16.45(-1.2,46.24) | 1.66(1.64,1.69) | 281.26(-23.03,832.15) | 118.04(-9.57,349.27) | 1007.7(-79.43,2947.38) | 175.18(-13.59,512.89) | 1.25(1.23,1.27) |
| Republic of Botswana | 91.96(-7.25,260.53) | 140.78(-10.91,400.08) | 399.67(-33.02,1112.21) | 246.4(-20.04,685.24) | 1.80(1.77,1.83) | 7.77(-0.58,22.36) | 12.7(-0.93,36.64) | 40.07(-3,109.28) | 26(-1.95,70.72) | 2.34(2.31,2.37) | 84.19(-6.63,239.69) | 128.08(-9.92,365.58) | 359.59(-29.95,1001.27) | 220.40(-18.05,612.87) | 1.74(1.70,1.78) |
| Republic of Bulgaria | 3573.95(-352.46,9949.65) | 215.34(-21.04,599.54) | 4843.12(-497.08,13242.34) | 247.06(-25.35,673.91) | 0.44(0.42,0.45) | 468.63(-42.4,1254.63) | 30.29(-2.72,81.39) | 678.91(-62.72,1782.04) | 34.81(-3.24,91.34) | 0.44(0.40,0.48) | 3105.32(-306.83,8655.5) | 185.05(-18.13,515.45) | 4164.21(-429.46,11471.88) | 212.26(-21.92,583.95) | 0.43(0.42,0.45) |
| Republic of Burundi | 168.06(-11.48,502.62) | 62.27(-4.23,186.88) | 460.46(-33.47,1377.96) | 85.21(-6.02,254.76) | 0.98(0.92,1.04) | 13.7(-0.88,41) | 5.32(-0.34,15.96) | 37.77(-2.52,113.82) | 7.47(-0.49,22.26) | 1.06(1.00,1.12) | 154.35(-10.61,457.72) | 56.95(-3.89,169.31) | 422.69(-30.98,1268.88) | 77.73(-5.54,233.38) | 0.97(0.91,1.03) |
| Republic of Cabo Verde | 38.68(-2.99,111.2) | 132.29(-10.31,380.23) | 117.6(-10.27,331.99) | 221.08(-18.94,626.47) | 1.68(1.67,1.69) | 3.03(-0.21,8.43) | 10.29(-0.73,28.59) | 10.12(-0.77,28.09) | 19.32(-1.45,53.53) | 2.07(2.06,2.09) | 35.65(-2.77,102.86) | 122.00(-9.58,351.98) | 107.48(-9.52,302.46) | 201.76(-17.52,570.01) | 1.65(1.63,1.66) |
| Republic of Cameroon | 955.27(-82.66,2770.53) | 189.22(-16.05,547.18) | 3669.87(-379.09,9955.81) | 269.63(-27.17,731.13) | 1.12(1.11,1.13) | 78.76(-6.25,222.22) | 16.48(-1.3,46.64) | 321.2(-30.3,849.36) | 24.98(-2.31,66.19) | 1.32(1.29,1.34) | 876.5(-76.26,2527.15) | 172.74(-14.72,497.41) | 3348.68(-346.94,9125.49) | 244.65(-24.75,664.97) | 1.10(1.09,1.12) |
| Republic of Chad | 330.49(-23.43,955.53) | 98.31(-6.95,283.98) | 813.98(-61.99,2357.38) | 128.01(-9.61,373.95) | 0.83(0.80,0.86) | 23.42(-1.54,68.78) | 7.21(-0.47,21.08) | 60.53(-4.19,180.01) | 10.08(-0.69,29.99) | 1.07(1.05,1.10) | 307.07(-21.86,886.56) | 91.10(-6.47,262.77) | 753.45(-57.66,2169.59) | 117.93(-8.90,342.62) | 0.81(0.78,0.84) |
| Republic of Chile | 3602.58(-366.23,9751.55) | 293.54(-29.54,795.27) | 12738.29(-1371.7,32895.34) | 384.08(-41.36,991.87) | 0.84(0.78,0.89) | 420.08(-44.15,1146.36) | 34.46(-3.59,94.07) | 1705.2(-180.14,4465.26) | 51.4(-5.43,134.63) | 1.28(1.17,1.39) | 3182.49(-322.23,8628.28) | 259.08(-25.97,702.89) | 11033.09(-1183.05,28808.86) | 332.67(-35.67,868.70) | 0.77(0.72,0.82) |
| Republic of Colombia | 4142.56(-388.32,11928.23) | 205.02(-19.08,590.25) | 20618.8(-2062.77,56581.02) | 296.81(-29.68,814.77) | 1.22(1.19,1.24) | 256.71(-22.2,715.19) | 13.03(-1.12,36.27) | 1397.1(-134.08,3751.73) | 19.99(-1.92,53.74) | 1.38(1.35,1.41) | 3885.85(-366.31,11192.27) | 192.00(-17.97,552.95) | 19221.71(-1928.02,52874.4) | 276.82(-27.75,761.59) | 1.20(1.18,1.23) |
| Republic of Costa Rica | 488.28(-47.53,1379.81) | 233.89(-22.64,661.04) | 2164.68(-222.41,5909.55) | 310.47(-31.77,847.62) | 0.91(0.89,0.94) | 32.09(-2.89,88) | 15.51(-1.39,42.54) | 151.25(-14.65,399.38) | 21.68(-2.1,57.24) | 1.07(1.04,1.10) | 456.19(-44.52,1291.06) | 218.38(-21.19,617.99) | 2013.43(-207.2,5516.82) | 288.79(-29.59,791.30) | 0.90(0.88,0.93) |
| Republic of Croatia | 1592.87(-156.41,4381.38) | 205.78(-19.81,566.61) | 3107.14(-312.83,8355.24) | 257.46(-26.24,691.69) | 0.77(0.74,0.81) | 202.83(-18.41,556.32) | 27.37(-2.43,75.13) | 444.59(-42.57,1163.74) | 36.36(-3.53,95.07) | 1.01(0.97,1.05) | 1390.03(-137.53,3840.31) | 178.41(-17.31,493.64) | 2662.55(-270.33,7230.23) | 221.10(-22.72,599.50) | 0.74(0.70,0.77) |
| Republic of Cuba | 2434.74(-202.07,7109.18) | 188.83(-15.69,550.8) | 6906.81(-653.97,19223.83) | 283.84(-27.1,788.96) | 1.40(1.36,1.43) | 170.58(-12.9,469.1) | 13.32(-1.01,36.63) | 525.64(-45.55,1400.31) | 21.24(-1.85,56.51) | 1.66(1.60,1.71) | 2264.16(-189.3,6576.26) | 175.51(-14.69,509.33) | 6381.17(-605.1,17863.49) | 262.60(-25.10,734.24) | 1.37(1.35,1.40) |
| Republic of Cyprus | 228.24(-19.05,649.67) | 208.05(-17.54,593.01) | 821.11(-82.3,2245.16) | 298.02(-29.94,814.27) | 1.18(1.12,1.24) | 30.41(-2.42,83.94) | 27.91(-2.25,77.21) | 121.93(-12.28,335.44) | 44.31(-4.47,121.72) | 1.55(1.47,1.63) | 197.82(-16.56,569.13) | 180.14(-15.21,519.03) | 699.18(-69.64,1920.65) | 253.71(-25.33,697.11) | 1.12(1.06,1.17) |
| Republic of Côte d'Ivoire | 528.71(-40.48,1539.72) | 126.38(-9.56,368.24) | 2274.66(-184.88,6489.9) | 192.28(-15.36,549.32) | 1.33(1.31,1.35) | 40.31(-2.75,115.98) | 10.45(-0.71,29.82) | 185.43(-14.89,511.66) | 16.7(-1.32,46.51) | 1.50(1.48,1.52) | 488.4(-37.43,1420) | 115.93(-8.79,336.94) | 2089.23(-169.74,5950.68) | 175.57(-14.02,500.83) | 1.31(1.30,1.33) |
| Republic of Djibouti | 8.46(-0.67,24.91) | 62.28(-4.93,185.63) | 66.83(-5.29,197.74) | 99.54(-7.73,296.16) | 1.54(1.50,1.58) | 0.69(-0.05,2.05) | 5.5(-0.42,16.45) | 6.15(-0.46,17.73) | 9.81(-0.73,28.25) | 1.94(1.88,2.01) | 7.77(-0.61,23.06) | 56.78(-4.49,170.02) | 60.68(-4.82,179.88) | 89.74(-6.99,268.16) | 1.50(1.46,1.53) |
| Republic of Ecuador | 1412.91(-126.1,3941.07) | 228.97(-20.45,639.16) | 6871.38(-687.48,18628.09) | 339.14(-33.86,919.57) | 1.34(1.30,1.37) | 102.73(-8.76,283.58) | 16.92(-1.44,46.74) | 532.11(-50.81,1391.06) | 26.5(-2.52,69.27) | 1.41(1.37,1.45) | 1310.18(-116.59,3670.78) | 212.05(-18.89,594.48) | 6339.28(-637.82,17247.05) | 312.64(-31.39,850.58) | 1.33(1.30,1.37) |
| Republic of El Salvador | 855.9(-85.88,2398.37) | 241.86(-24.11,677.8) | 2500.47(-265.38,6709.75) | 325.92(-34.79,874.01) | 0.96(0.91,1.01) | 54.72(-5.3,150.07) | 15.54(-1.49,42.61) | 173.32(-17.76,456.07) | 22.09(-2.28,58.05) | 1.12(1.03,1.20) | 801.19(-80.3,2244.08) | 226.32(-22.54,634.13) | 2327.15(-247.15,6205.73) | 303.83(-32.45,810.16) | 0.95(0.90,1.00) |
| Republic of Equatorial Guinea | 24.79(-1.86,72.18) | 112.42(-8.41,326.55) | 121.87(-10.75,347.92) | 232.21(-19.95,663.53) | 2.51(2.45,2.56) | 1.99(-0.14,5.67) | 9.62(-0.65,27.44) | 11.99(-0.96,32.46) | 24.08(-1.9,65.33) | 3.28(3.17,3.39) | 22.8(-1.71,66.53) | 102.80(-7.69,299.49) | 109.88(-9.72,313.69) | 208.12(-17.95,593.69) | 2.43(2.37,2.48) |
| Republic of Estonia | 601.13(-54.93,1640.81) | 226.2(-20.75,617.79) | 989.05(-101.78,2619.34) | 275.01(-28.88,726.51) | 0.72(0.69,0.75) | 72.39(-6.06,193.76) | 27.83(-2.33,74.69) | 135.46(-12.87,359.02) | 36.31(-3.52,96.24) | 1.00(0.96,1.05) | 528.73(-48.65,1442.45) | 198.37(-18.34,541.49) | 853.59(-88.69,2268.55) | 238.70(-25.31,632.79) | 0.68(0.65,0.70) |
| Republic of Fiji | 83.95(-8.13,232.36) | 226.42(-21.52,627.81) | 287.93(-35.59,753.66) | 315.35(-37.38,827) | 1.03(0.96,1.09) | 6.4(-0.6,17.22) | 18.18(-1.68,49.04) | 22.26(-2.5,58.19) | 25.8(-2.77,67.44) | 1.05(0.94,1.15) | 77.54(-7.52,215.95) | 208.23(-19.82,579.64) | 265.67(-33.04,696.11) | 289.55(-34.55,760.37) | 1.02(0.96,1.08) |
| Republic of Finland | 2492.76(-226.83,6923.9) | 263.86(-24.11,733.56) | 5450.07(-559.83,14603.76) | 322.85(-33.63,864.8) | 0.63(0.58,0.67) | 356.56(-31.06,963.24) | 37.77(-3.3,101.95) | 852.76(-85.83,2246.22) | 50.05(-5.08,131.69) | 0.90(0.83,0.97) | 2136.2(-195.05,5977.09) | 226.09(-20.74,632.84) | 4597.31(-468.87,12334.97) | 272.80(-28.22,732.31) | 0.58(0.54,0.62) |
| Republic of Ghana | 723.81(-55.95,2209.24) | 103.29(-7.95,314.78) | 3844.86(-300.11,11031.97) | 204.85(-15.71,587.89) | 2.23(2.22,2.24) | 54.45(-3.75,157.59) | 8.29(-0.57,24.1) | 319.02(-25.09,882.01) | 18.06(-1.37,50.07) | 2.56(2.53,2.58) | 669.36(-52.12,2047.35) | 95.00(-7.36,290.29) | 3525.84(-274.92,10142.34) | 186.79(-14.33,537.48) | 2.20(2.19,2.21) |
| Republic of Guatemala | 797.94(-68.99,2253.69) | 203.26(-17.69,572.28) | 3688.74(-350.56,10010.98) | 276.8(-26.35,750.48) | 1.02(1.01,1.03) | 45.49(-3.76,123.47) | 12.19(-1.02,32.98) | 230.66(-22.43,617.16) | 17.65(-1.72,47.14) | 1.21(1.20,1.23) | 752.45(-65.12,2117.84) | 191.07(-16.65,536.44) | 3458.08(-327.7,9344.63) | 259.15(-24.60,699.66) | 1.00(0.99,1.01) |
| Republic of Guinea | 439.17(-39.15,1264.01) | 106.42(-9.38,307.06) | 976.33(-80.44,2899.18) | 150.55(-12.22,448.1) | 1.08(1.05,1.12) | 32.36(-2.49,94.29) | 8.18(-0.62,23.85) | 76.48(-5.74,219.72) | 12.3(-0.91,35.41) | 1.32(1.27,1.37) | 406.81(-36.57,1166.03) | 98.24(-8.73,282.27) | 899.85(-74.84,2667.1) | 138.25(-11.33,411.29) | 1.06(1.03,1.09) |
| Republic of Guinea-Bissau | 48.91(-4.03,141.96) | 107.69(-8.95,313.55) | 123.37(-10.63,351.96) | 160.45(-13.46,457.1) | 1.25(1.23,1.27) | 3.5(-0.26,10.11) | 8.27(-0.62,23.78) | 9.16(-0.72,26.02) | 12.86(-0.99,36.52) | 1.37(1.34,1.40) | 45.4(-3.77,131.29) | 99.42(-8.34,287.71) | 114.21(-9.89,326.58) | 147.59(-12.44,422.21) | 1.24(1.22,1.26) |
| Republic of Guyana | 85.46(-7.15,242.16) | 193.68(-16.15,549.08) | 206.53(-20.33,579.55) | 265.8(-25.53,747.86) | 1.05(1.02,1.07) | 5.78(-0.48,16.11) | 13.52(-1.11,37.6) | 14.57(-1.32,38.91) | 19.51(-1.73,52.45) | 1.21(1.17,1.24) | 79.68(-6.69,225.29) | 180.16(-15.06,509.09) | 191.97(-18.94,540.39) | 246.29(-23.71,694.41) | 1.04(1.01,1.06) |
| Republic of Haiti | 313.19(-21.95,926.83) | 82.67(-5.87,245.47) | 1105.77(-88.95,3154.7) | 136.05(-10.72,388.1) | 1.73(1.69,1.78) | 18.62(-1.27,55.7) | 5.19(-0.36,15.53) | 67.19(-4.92,189.42) | 8.73(-0.63,24.72) | 1.86(1.80,1.92) | 294.57(-20.72,869.69) | 77.48(-5.52,229.57) | 1038.58(-83.49,2972.21) | 127.32(-10.04,364.32) | 1.72(1.68,1.77) |
| Republic of Honduras | 495.67(-44.19,1388.29) | 210.01(-18.6,589.31) | 2099(-227.29,5617.92) | 278.19(-29.7,745.45) | 0.91(0.89,0.93) | 29.61(-2.56,81.66) | 12.92(-1.11,35.6) | 131.94(-12.86,344.8) | 18.12(-1.73,47.51) | 1.12(1.07,1.17) | 466.06(-41.61,1306.36) | 197.10(-17.48,552.89) | 1967.06(-213.78,5262.76) | 260.06(-27.89,696.69) | 0.90(0.88,0.92) |
| Republic of Iceland | 107.3(-10.46,296.52) | 288.82(-28.22,798.96) | 261.6(-26.27,701.94) | 343.57(-34.68,921.14) | 0.54(0.52,0.56) | 18.9(-1.79,51.16) | 50.78(-4.83,137.4) | 44.64(-4.47,119.24) | 58.36(-5.87,155.92) | 0.30(0.20,0.40) | 88.4(-8.67,246.85) | 238.04(-23.38,665.57) | 216.96(-21.76,588.44) | 285.21(-28.75,773.18) | 0.59(0.57,0.61) |
| Republic of India | 26607.33(-1757.47,81556.72) | 51.82(-3.41,158.22) | 167341.36(-12220.05,484301.52) | 114.96(-8.35,334.24) | 2.74(2.70,2.79) | 1547.22(-98.61,4486.34) | 3.14(-0.2,9.08) | 11403.03(-778.14,32032.64) | 8.04(-0.55,22.65) | 3.36(3.25,3.48) | 25060.11(-1661.77,76960.05) | 48.68(-3.21,148.88) | 155938.32(-11427.14,453178.6) | 106.92(-7.79,312.21) | 2.70(2.65,2.75) |
| Republic of Indonesia | 5299.41(-402.69,15942.44) | 48.05(-3.62,145.32) | 27647.86(-2168.29,79283.13) | 95.99(-7.34,276.64) | 2.42(2.36,2.48) | 407.53(-28.03,1184.83) | 3.92(-0.27,11.49) | 2157.3(-160.03,6186.62) | 7.92(-0.57,22.67) | 2.46(2.40,2.52) | 4891.88(-375.13,14837.2) | 44.13(-3.35,134.56) | 25490.57(-2020.9,73225.64) | 88.07(-6.80,254.63) | 2.42(2.36,2.47) |
| Republic of Iraq | 2058.21(-185.9,5623.49) | 228.73(-20.67,624.9) | 7031.11(-792.72,18942.33) | 271.46(-30.11,731.67) | 0.58(0.56,0.59) | 136(-11.66,369.84) | 15.27(-1.31,41.57) | 469.23(-50.05,1222.91) | 19.14(-2.02,49.93) | 0.77(0.73,0.81) | 1922.21(-173.97,5223.35) | 213.46(-19.32,579.97) | 6561.88(-741.57,17661.94) | 252.32(-28.05,679.49) | 0.56(0.54,0.58) |
| Republic of Italy | 28338.06(-2415.85,80599.7) | 238.08(-20.39,676.59) | 55202.71(-5124.43,152298.66) | 297.69(-27.84,821.76) | 0.68(0.65,0.71) | 3854.76(-332.54,10461.93) | 32.42(-2.81,87.91) | 8105.71(-758.1,21792.24) | 43.16(-4.08,115.63) | 1.04(0.97,1.11) | 24483.3(-2081.33,69923.81) | 205.65(-17.56,586.52) | 47097.01(-4348.62,131410.86) | 254.53(-23.67,710.77) | 0.62(0.58,0.66) |
| Republic of Kazakhstan | 2640.35(-245.21,7402.25) | 176.42(-16.2,495.53) | 4665.05(-456.23,12767.91) | 217.06(-20.95,593.54) | 0.67(0.65,0.68) | 376.56(-32.04,1028.49) | 26.05(-2.21,71.02) | 693.96(-64.29,1816.21) | 33.94(-3.15,88.96) | 0.82(0.80,0.85) | 2263.79(-208.49,6255.44) | 150.37(-13.72,416.03) | 3971.09(-387.96,10803.89) | 183.11(-17.64,497.65) | 0.64(0.63,0.65) |
| Republic of Kenya | 851.65(-67.08,2463.54) | 90.58(-7.03,261.27) | 4163.93(-337.51,12123.41) | 159.54(-12.7,465.14) | 1.85(1.80,1.90) | 78.16(-5.69,220.05) | 8.72(-0.63,24.52) | 400.54(-29.66,1116.91) | 16.25(-1.19,45.34) | 2.07(1.98,2.15) | 773.49(-61.42,2257.57) | 81.86(-6.40,238.48) | 3763.39(-306.31,10956.75) | 143.28(-11.45,417.39) | 1.83(1.79,1.87) |
| Republic of Kiribati | 8.37(-0.84,23.62) | 196.78(-19.34,554.76) | 22.45(-2.32,60.52) | 271.6(-27.28,735.06) | 1.00(0.92,1.08) | 0.59(-0.05,1.6) | 14.63(-1.32,39.55) | 1.56(-0.15,4.12) | 20.17(-1.91,53.34) | 0.93(0.78,1.08) | 7.78(-0.79,21.89) | 182.15(-17.98,511.98) | 20.89(-2.16,56.6) | 251.43(-25.31,683.33) | 1.01(0.93,1.08) |
| Republic of Korea | 6392.63(-568.16,18334.95) | 193.53(-17.14,554.78) | 36048.81(-2951.91,102917.64) | 288.7(-23.57,824.91) | 1.52(1.36,1.67) | 421.77(-34.42,1202.08) | 12.86(-1.05,36.68) | 2619.27(-217.62,7299.3) | 21.04(-1.74,58.64) | 1.66(1.59,1.73) | 5970.87(-532.88,17259.1) | 180.67(-16.06,521.82) | 33429.54(-2734.22,95400.59) | 267.67(-21.83,764.86) | 1.51(1.35,1.67) |
| Republic of Latvia | 1094.48(-110.91,2977.98) | 235.68(-23.89,641.05) | 1492.07(-164.51,4014.6) | 279.52(-31.1,752.24) | 0.59(0.57,0.61) | 133.15(-13.04,351.91) | 29.16(-2.86,77.19) | 202.15(-20.77,531.94) | 36.71(-3.8,96.68) | 0.82(0.79,0.85) | 961.33(-97.44,2587.48) | 206.52(-20.95,555.50) | 1289.92(-143,3503.95) | 242.81(-27.16,659.18) | 0.56(0.54,0.58) |
| Republic of Liberia | 226.03(-18.66,657.59) | 157.68(-12.96,458.67) | 504.62(-45.64,1423.71) | 230.97(-20.57,652.02) | 1.32(1.27,1.38) | 17.77(-1.36,49.55) | 12.91(-0.98,36.21) | 42.41(-3.33,117.14) | 20.33(-1.58,56.28) | 1.67(1.56,1.77) | 208.26(-17.24,609.87) | 144.77(-11.94,423.75) | 462.21(-42.07,1297.23) | 210.65(-18.87,591.51) | 1.29(1.24,1.34) |
| Republic of Lithuania | 1277.65(-123.22,3507.68) | 220.86(-21.33,606.9) | 2115.1(-212.27,5624.97) | 275.17(-28.09,730.7) | 0.72(0.69,0.76) | 156.56(-13.9,431.51) | 27.42(-2.43,75.54) | 283.22(-25.99,758.57) | 35.64(-3.34,95.23) | 0.91(0.86,0.95) | 1121.09(-108.64,3069.08) | 193.44(-18.80,529.88) | 1831.89(-185.66,4806.92) | 239.53(-24.67,628.30) | 0.70(0.66,0.73) |
| Republic of Madagascar | 409.93(-28.32,1237.14) | 70.55(-4.86,211.17) | 1383.82(-106.5,3918.89) | 118.75(-8.83,338.15) | 1.71(1.67,1.75) | 34.01(-2.34,100.85) | 6.2(-0.42,18.22) | 113.72(-8.01,326.19) | 10.57(-0.73,30.19) | 1.78(1.71,1.84) | 375.91(-25.88,1139.59) | 64.35(-4.41,193.66) | 1270.1(-98.43,3592.69) | 108.18(-8.10,307.95) | 1.70(1.66,1.74) |
| Republic of Malawi | 344.58(-30.39,1019.34) | 79.54(-6.8,236.96) | 1081.24(-83.88,3137.81) | 131.39(-10.23,380.19) | 1.67(1.64,1.70) | 29.84(-2.17,86.05) | 7.47(-0.53,21.57) | 99.34(-7.68,282.26) | 12.8(-0.98,36.3) | 1.81(1.77,1.85) | 314.75(-27.94,938.81) | 72.07(-6.21,216.39) | 981.9(-76.37,2840.88) | 118.58(-9.26,342.65) | 1.66(1.63,1.69) |
| Republic of Maldives | 7.29(-0.59,21.43) | 66.64(-5.33,196.92) | 51.42(-4.53,146.09) | 138.23(-11.76,396.69) | 2.60(2.52,2.68) | 0.54(-0.04,1.52) | 5.28(-0.39,15.09) | 4.1(-0.31,11.51) | 11.39(-0.86,32.11) | 2.78(2.67,2.88) | 6.75(-0.55,19.92) | 61.36(-4.94,182.03) | 47.32(-4.17,134.69) | 126.84(-10.81,364.05) | 2.59(2.51,2.67) |
| Republic of Mali | 455.54(-35.01,1339.05) | 97.86(-7.26,289.44) | 1289.26(-94.21,3819.32) | 128.25(-9.31,376.95) | 0.87(0.82,0.92) | 31.83(-2.28,93.19) | 7.33(-0.51,21.4) | 97.17(-6.77,283.04) | 10.29(-0.71,29.73) | 1.10(1.05,1.15) | 423.71(-32.71,1248.67) | 90.53(-6.75,268.07) | 1192.09(-87,3528.23) | 117.97(-8.56,346.75) | 0.85(0.80,0.90) |
| Republic of Malta | 124.68(-10.68,353.7) | 226.43(-19.37,642.31) | 416.39(-40.76,1132.53) | 312.52(-30.99,848.33) | 1.01(0.90,1.11) | 17.22(-1.42,48.07) | 31.4(-2.59,87.74) | 64.43(-6.05,174.03) | 48.07(-4.58,129.61) | 1.32(1.17,1.47) | 107.45(-9.18,302.23) | 195.03(-16.64,548.54) | 351.96(-34.38,959.4) | 264.45(-26.17,720.69) | 0.95(0.86,1.05) |
| Republic of Mauritius | 107.1(-8.64,306.87) | 118.54(-9.53,341.19) | 453.18(-37.67,1245.17) | 184.17(-15.06,507.98) | 1.47(1.42,1.52) | 8.47(-0.62,23.94) | 9.71(-0.71,27.42) | 37.95(-3.01,102.49) | 15.78(-1.23,42.67) | 1.63(1.58,1.68) | 98.63(-8.02,281.13) | 108.82(-8.82,311.95) | 415.23(-34.63,1137.56) | 168.39(-13.82,462.68) | 1.46(1.41,1.51) |
| Republic of Moldova | 1189.58(-111.11,3284.7) | 215.83(-19.79,597.99) | 2258.67(-234.3,5990.16) | 281.82(-29.07,747.59) | 0.92(0.89,0.95) | 133.82(-11.59,356.7) | 25.86(-2.18,69.25) | 290.69(-27.31,767.99) | 36.49(-3.42,96.35) | 1.24(1.17,1.30) | 1055.75(-99.42,2903.92) | 189.97(-17.56,524.03) | 1967.98(-206.12,5206.83) | 245.34(-25.54,649.24) | 0.88(0.85,0.90) |
| Republic of Mozambique | 530.35(-41.18,1586.1) | 77.45(-6,232.17) | 1585.81(-114.41,4704.56) | 127.77(-9.07,379.7) | 1.67(1.60,1.73) | 43.08(-2.96,123.01) | 6.67(-0.47,19.07) | 134.95(-9.34,382.21) | 11.48(-0.79,32.6) | 1.82(1.75,1.89) | 487.27(-38.25,1461.24) | 70.78(-5.53,212.56) | 1450.86(-104.82,4341.61) | 116.29(-8.26,348.12) | 1.65(1.59,1.71) |
| Republic of Namibia | 105.72(-7.89,309.25) | 138.26(-10.2,407.1) | 354.67(-28.18,1012.1) | 231.46(-18.21,661.09) | 1.68(1.63,1.74) | 9.45(-0.64,27.9) | 13.16(-0.89,38.86) | 34.08(-2.52,93.28) | 23.39(-1.71,64.09) | 1.90(1.83,1.96) | 96.27(-7.23,281.55) | 125.10(-9.29,366.65) | 320.59(-25.46,910.46) | 208.06(-16.38,590.13) | 1.66(1.60,1.72) |
| Republic of Nauru | 1.23(-0.13,3.27) | 233.67(-24.23,628.04) | 2.06(-0.25,5.34) | 316.88(-37,827.78) | 0.94(0.91,0.96) | 0.1(-0.01,0.26) | 19.74(-1.96,53.11) | 0.15(-0.02,0.4) | 25.08(-2.73,65.53) | 0.64(0.58,0.70) | 1.14(-0.12,3.01) | 213.93(-22.14,573.35) | 1.91(-0.23,4.97) | 291.79(-34.21,764.92) | 0.97(0.94,0.99) |
| Republic of Nicaragua | 419.93(-38.59,1154.73) | 240.96(-21.99,664.52) | 1824.9(-210.13,4865.5) | 316.25(-36.2,843.33) | 0.88(0.85,0.92) | 25.49(-2.23,69.74) | 14.95(-1.3,40.89) | 118.42(-12.3,312.96) | 20.89(-2.16,55.3) | 1.07(1.01,1.14) | 394.43(-36.37,1085.91) | 226.02(-20.70,623.81) | 1706.47(-197.6,4553.82) | 295.36(-33.99,788.10) | 0.87(0.84,0.90) |
| Republic of Niue | 0.64(-0.06,1.77) | 232.88(-22.53,639.87) | 0.89(-0.1,2.35) | 317.23(-34.15,837.47) | 1.01(0.95,1.06) | 0.05(0,0.14) | 18.9(-1.71,50.85) | 0.08(-0.01,0.2) | 27.17(-2.69,71.69) | 1.11(0.99,1.24) | 0.59(-0.06,1.63) | 213.99(-20.81,592.12) | 0.82(-0.09,2.17) | 290.06(-31.25,769.36) | 1.00(0.94,1.05) |
| Republic of Palau | 3.06(-0.31,8.19) | 253.76(-24.84,680.01) | 8.98(-1.04,23.57) | 321.73(-35.48,847.67) | 0.71(0.64,0.78) | 0.25(-0.02,0.67) | 21.89(-1.92,57.68) | 0.74(-0.08,1.91) | 28.74(-2.85,74.25) | 0.74(0.61,0.87) | 2.81(-0.28,7.52) | 231.88(-22.91,622.34) | 8.23(-0.96,21.77) | 292.99(-32.62,776.36) | 0.71(0.65,0.77) |
| Republic of Panama | 444.37(-42.86,1222.9) | 251.04(-24.09,691.73) | 1855.41(-213.3,4837.75) | 338.87(-39,883.42) | 0.94(0.93,0.96) | 27.46(-2.4,72.78) | 15.69(-1.37,41.63) | 128.01(-13.68,336.66) | 23.18(-2.48,60.98) | 1.20(1.17,1.23) | 416.91(-40.39,1149.51) | 235.35(-22.68,649.81) | 1727.41(-198.53,4525.64) | 315.69(-36.32,827.14) | 0.93(0.91,0.94) |
| Republic of Paraguay | 602.65(-58.42,1699.49) | 228.25(-22.01,644.11) | 2113.9(-233.12,5717.44) | 298.56(-32.54,808.49) | 0.90(0.89,0.91) | 41.81(-3.62,114.33) | 16.05(-1.39,43.93) | 155.41(-15.73,412.86) | 22.25(-2.23,59.12) | 1.10(1.08,1.12) | 560.84(-54.46,1578.45) | 212.20(-20.49,597.76) | 1958.5(-216.91,5270.48) | 276.30(-30.24,744.31) | 0.88(0.87,0.90) |
| Republic of Peru | 2898.66(-263.7,8144.44) | 209.07(-18.92,588.85) | 11820.35(-1211.88,32459.57) | 290.92(-29.78,799.19) | 1.09(1.05,1.13) | 197.2(-17.35,546.8) | 14.4(-1.26,39.93) | 874.32(-81.41,2335.83) | 21.46(-2,57.32) | 1.36(1.30,1.41) | 2701.46(-245.25,7605.37) | 194.67(-17.58,549.20) | 10946.03(-1128.17,29862.28) | 269.46(-27.73,735.35) | 1.07(1.03,1.11) |
| Republic of Poland | 11844.74(-1089.54,32535.87) | 209.03(-19.22,574.64) | 25128.92(-2584.81,67537.75) | 255.04(-26.22,685.31) | 0.65(0.63,0.67) | 1610.41(-142.67,4322.61) | 29.34(-2.59,79) | 3793.1(-369.89,9949.37) | 38.29(-3.73,100.38) | 0.91(0.89,0.92) | 10234.33(-946.47,28080.49) | 179.69(-16.62,493.51) | 21335.82(-2203.8,57436.12) | 216.75(-22.37,583.48) | 0.61(0.59,0.63) |
| Republic of Rwanda | 250.6(-19.73,758.95) | 76.46(-5.97,232.54) | 899.36(-70.46,2592.1) | 122.59(-9.55,354.37) | 1.56(1.52,1.59) | 20.4(-1.41,57.9) | 6.67(-0.47,19.1) | 76.25(-5.57,221.06) | 11.04(-0.8,32.13) | 1.69(1.62,1.76) | 230.21(-18.28,700.56) | 69.79(-5.49,212.86) | 823.11(-64.85,2366.35) | 111.55(-8.74,321.35) | 1.54(1.51,1.57) |
| Republic of San Marino | 12.81(-1.17,35.25) | 275.81(-25.47,758.57) | 30.97(-3.05,83.38) | 332.38(-33.49,894.38) | 0.59(0.55,0.62) | 1.89(-0.17,5.04) | 40.61(-3.7,108.38) | 4.93(-0.49,13.04) | 52.15(-5.27,137.71) | 0.80(0.74,0.86) | 10.92(-0.99,30.07) | 235.20(-21.64,648.71) | 26.04(-2.56,70.7) | 280.23(-28.14,761.61) | 0.55(0.52,0.58) |
| Republic of Senegal | 482.79(-39.46,1401.83) | 127.33(-10.25,369.6) | 1610.9(-129.15,4615.71) | 179.26(-14.24,515) | 1.07(1.04,1.09) | 37.88(-2.8,107) | 10.48(-0.76,29.68) | 130.38(-9.55,363.85) | 15.19(-1.12,42.44) | 1.17(1.13,1.20) | 444.91(-36.45,1293.38) | 116.85(-9.43,338.92) | 1480.52(-119.9,4257.94) | 164.07(-13.14,472.64) | 1.06(1.03,1.08) |
| Republic of Serbia | 2888.35(-275.29,7897.21) | 198.79(-18.72,544.69) | 5870.44(-615.48,15500.7) | 259.63(-27.33,685.77) | 0.91(0.89,0.92) | 364.1(-30.66,997.39) | 26.72(-2.21,72.91) | 825.65(-83.12,2149.96) | 36.54(-3.69,95.32) | 1.12(1.09,1.15) | 2524.24(-244.3,6913.7) | 172.07(-16.45,473.25) | 5044.78(-532.89,13407.79) | 223.09(-23.67,592.68) | 0.87(0.86,0.89) |
| Republic of Seychelles | 10.6(-0.92,30.41) | 145.89(-12.74,418.76) | 30.43(-3.33,82.22) | 211.97(-22.53,575.87) | 1.19(1.14,1.24) | 0.87(-0.07,2.4) | 11.93(-0.9,33.08) | 2.59(-0.25,6.99) | 18.59(-1.74,50.44) | 1.39(1.35,1.42) | 9.73(-0.86,28.06) | 133.96(-11.80,386.32) | 27.84(-3.06,75.43) | 193.38(-20.67,526.47) | 1.17(1.12,1.23) |
| Republic of Sierra Leone | 271.09(-19.96,804.34) | 110.44(-8.05,328.2) | 668.73(-52.21,1891.13) | 159.13(-12.38,451.08) | 1.16(1.11,1.20) | 20.27(-1.45,58.64) | 8.55(-0.6,24.81) | 52.54(-3.94,146.15) | 13.06(-0.98,36.37) | 1.34(1.26,1.42) | 250.81(-18.49,747.22) | 101.89(-7.44,304.08) | 616.19(-48.3,1751.04) | 146.08(-11.39,415.96) | 1.14(1.10,1.18) |
| Republic of Singapore | 442.09(-36.39,1298.63) | 171.5(-14.09,503.34) | 3528.36(-282.47,9923.97) | 316.03(-25.09,888.9) | 1.98(1.90,2.06) | 31.53(-2.59,91.73) | 12.3(-1.01,35.7) | 281.27(-22.67,781.43) | 25.25(-2.02,70.13) | 2.38(2.28,2.47) | 410.56(-33.78,1215.81) | 159.20(-13.08,470.91) | 3247.09(-259.96,9146.33) | 290.78(-23.08,819.16) | 1.95(1.87,2.03) |
| Republic of Slovenia | 682.73(-63.84,1884.24) | 217.52(-20.27,600.89) | 1534.93(-152.65,4081.48) | 260.71(-26.4,692.89) | 0.61(0.60,0.62) | 90.6(-7.59,242.42) | 29.29(-2.46,78.46) | 229.35(-21.81,602.11) | 37.66(-3.64,98.73) | 0.85(0.83,0.88) | 592.13(-55.94,1630.26) | 188.23(-17.72,518.97) | 1305.58(-130.95,3475.39) | 223.05(-22.77,593.55) | 0.57(0.56,0.58) |
| Republic of South Africa | 5434.45(-504.45,15059.93) | 223.52(-20.43,620.97) | 16565.59(-1675.55,44525.94) | 297.98(-29.84,802.19) | 0.95(0.94,0.96) | 556.65(-47.1,1493.96) | 23.51(-1.96,63.3) | 1779.54(-170.32,4564.95) | 33.24(-3.15,85.66) | 1.22(1.19,1.24) | 4877.8(-454.25,13389.18) | 200.01(-18.35,550.33) | 14786.05(-1497.22,40014.53) | 264.74(-26.55,717.03) | 0.92(0.91,0.93) |
| Republic of South Sudan | 177.76(-13.97,536.23) | 58.15(-4.54,175.67) | 325.97(-25.98,947.37) | 81.5(-6.32,238.76) | 1.13(1.08,1.19) | 15.62(-1.17,45.92) | 5.36(-0.4,15.8) | 29.05(-2.06,82.89) | 7.71(-0.53,21.95) | 1.21(1.14,1.27) | 162.14(-12.78,490.44) | 52.79(-4.13,159.57) | 296.92(-23.72,863.37) | 73.79(-5.74,215.66) | 1.12(1.07,1.18) |
| Republic of Sudan | 1567.21(-131.79,4544.05) | 145.06(-12.02,420.57) | 4987.29(-499.7,13669.2) | 237.71(-23.42,653.37) | 1.64(1.60,1.69) | 83.99(-6.72,234.14) | 8.19(-0.64,22.88) | 321.14(-29.39,852.89) | 15.96(-1.44,42.67) | 2.23(2.17,2.30) | 1483.22(-124.82,4313.07) | 136.87(-11.35,397.60) | 4666.16(-470.16,12853.86) | 221.74(-21.98,612.01) | 1.60(1.56,1.64) |
| Republic of Suriname | 52.87(-4.7,152.29) | 175.4(-15.38,505.98) | 198.77(-17.88,552.95) | 254.8(-22.72,709.32) | 1.25(1.23,1.26) | 3.78(-0.3,10.68) | 12.75(-1.02,35.95) | 14.78(-1.26,39.9) | 19.22(-1.62,51.88) | 1.40(1.38,1.42) | 49.09(-4.4,141.17) | 162.65(-14.37,468.51) | 183.99(-16.59,509.78) | 235.58(-21.05,653.32) | 1.24(1.22,1.25) |
| Republic of Tajikistan | 479.03(-41.98,1371.64) | 151.2(-13.07,433.24) | 1097.91(-113.52,3048.56) | 174.25(-17.3,486.27) | 0.43(0.41,0.45) | 63.74(-5.1,174.9) | 20.64(-1.63,56.55) | 145.46(-13.45,390.53) | 24.77(-2.2,67.14) | 0.56(0.54,0.59) | 415.29(-36.87,1184.32) | 130.56(-11.44,372.58) | 952.45(-99.11,2620.55) | 149.48(-14.95,412.82) | 0.41(0.39,0.43) |
| Republic of the Congo | 142.83(-11.99,418.05) | 110.95(-9.34,324.83) | 534.59(-45.76,1500.85) | 185.69(-15.5,521.08) | 1.65(1.62,1.68) | 11.98(-0.95,34.47) | 9.89(-0.78,28.38) | 48.55(-4.08,133.3) | 17.8(-1.45,49.08) | 1.92(1.90,1.93) | 130.85(-11.04,384.58) | 101.06(-8.55,297.67) | 486.05(-41.45,1364.27) | 167.89(-13.99,471.67) | 1.62(1.59,1.65) |
| Republic of the Gambia | 52.7(-4.37,156.31) | 133.33(-10.85,395.97) | 210.24(-18.01,599.79) | 192.22(-16.22,549.87) | 1.16(1.14,1.19) | 3.93(-0.29,11.07) | 10.43(-0.75,29.42) | 17.04(-1.28,48.28) | 16.16(-1.2,45.99) | 1.40(1.37,1.44) | 48.77(-4.06,144.7) | 122.89(-10.04,365.35) | 193.2(-16.68,546.03) | 176.06(-14.97,498.58) | 1.14(1.12,1.16) |
| Republic of the Marshall Islands | 3.99(-0.4,10.97) | 213.74(-21.14,589.72) | 10.56(-1.2,27.74) | 270.08(-29.96,717.06) | 0.69(0.67,0.72) | 0.28(-0.03,0.75) | 15.63(-1.39,42.11) | 0.75(-0.08,1.94) | 20.98(-2.14,54.58) | 0.89(0.82,0.96) | 3.71(-0.38,10.17) | 198.11(-19.74,544.69) | 9.82(-1.13,25.86) | 249.10(-27.81,662.13) | 0.68(0.65,0.70) |
| Republic of the Niger | 289.35(-22.48,843.76) | 92.83(-7.18,271.33) | 1134.02(-81.22,3237.52) | 119.7(-8.47,342.26) | 0.85(0.83,0.87) | 20.13(-1.5,58.92) | 6.98(-0.52,20.52) | 80.98(-5.63,231.34) | 9.14(-0.63,26.02) | 0.90(0.88,0.93) | 269.22(-20.95,785.34) | 85.85(-6.66,251.06) | 1053.04(-75.6,2999.45) | 110.56(-7.84,315.88) | 0.84(0.82,0.86) |
| Republic of the Philippines | 2210.48(-154.12,6550.07) | 66.28(-4.6,196.91) | 11233.13(-805.08,33675.2) | 115.7(-8.2,347.31) | 1.82(1.71,1.94) | 199.04(-13.52,561.67) | 6.23(-0.42,17.6) | 971.34(-65.89,2809.35) | 10.4(-0.7,30.18) | 1.65(1.59,1.72) | 2011.44(-140.38,5997.94) | 60.04(-4.18,179.39) | 10261.79(-740.86,30628.45) | 105.30(-7.52,315.03) | 1.84(1.72,1.96) |
| Republic of the Union of Myanmar | 1607.44(-124.98,4859.42) | 56.7(-4.4,171.55) | 5629.88(-440.49,16382.27) | 92.81(-7.18,270.05) | 1.69(1.66,1.73) | 111.45(-7.87,330.06) | 4.14(-0.3,12.28) | 397.02(-28.36,1121.9) | 6.78(-0.48,19.19) | 1.72(1.66,1.78) | 1495.99(-117.12,4533.13) | 52.56(-4.10,159.37) | 5232.86(-412.68,15269.27) | 86.03(-6.70,251.05) | 1.69(1.66,1.72) |
| Republic of Trinidad and Tobago | 248.13(-23.66,700.93) | 237.26(-22.54,671.42) | 812.42(-87.29,2215.34) | 314.31(-33.47,858.97) | 0.95(0.93,0.98) | 17.95(-1.54,49.52) | 17.51(-1.49,48.35) | 62.05(-6.13,163.65) | 24.4(-2.38,64.52) | 1.18(1.14,1.22) | 230.18(-21.99,649.56) | 219.75(-20.93,620.83) | 750.36(-80.76,2047.73) | 289.91(-30.94,793.11) | 0.93(0.91,0.96) |
| Republic of Tunisia | 981.38(-90.11,2832.71) | 160.37(-14.46,463.79) | 4269.99(-463.57,11660.64) | 253.59(-27.08,693.05) | 1.52(1.50,1.53) | 56.99(-4.89,156.7) | 9.85(-0.83,27.08) | 284.19(-25.17,748.15) | 17.43(-1.53,46.03) | 1.88(1.86,1.89) | 924.38(-85.08,2674.89) | 150.51(-13.61,436.13) | 3985.8(-437.38,10908.43) | 236.16(-25.48,646.53) | 1.49(1.48,1.50) |
| Republic of Turkey | 8859.3(-886.11,24370.2) | 220.95(-21.66,608.16) | 36408.33(-4224.05,96419.59) | 308.91(-35.36,818.43) | 1.07(1.05,1.09) | 515.58(-46.33,1398.91) | 13.42(-1.19,36.45) | 2449.23(-272.39,6377.14) | 21.29(-2.33,55.52) | 1.43(1.38,1.48) | 8343.72(-835.25,22888.73) | 207.53(-20.35,569.73) | 33959.11(-3946.77,89955.93) | 287.62(-32.99,762.50) | 1.04(1.02,1.07) |
| Republic of Uganda | 583.98(-42.45,1772.34) | 77.6(-5.63,235.99) | 2057.77(-170.37,5879.92) | 127.17(-10.38,363.97) | 1.66(1.61,1.71) | 48.04(-3.48,140.78) | 6.68(-0.48,19.67) | 177.23(-14.27,501.57) | 11.44(-0.91,32.5) | 1.82(1.76,1.89) | 535.94(-38.9,1628.06) | 70.91(-5.14,215.94) | 1880.55(-155.8,5364.25) | 115.72(-9.45,330.87) | 1.64(1.59,1.69) |
| Republic of Uzbekistan | 2280.26(-216.39,6249.21) | 168.75(-15.86,463.64) | 6047.64(-668.07,16017.03) | 205.51(-21.55,547.97) | 0.65(0.64,0.66) | 316.02(-29.15,864.53) | 23.78(-2.17,65.01) | 864.17(-90.08,2286.35) | 31.08(-3.06,82.82) | 0.88(0.86,0.91) | 1964.23(-186.12,5398.52) | 144.97(-13.62,399.00) | 5183.46(-575.07,13710.49) | 174.42(-18.43,464.36) | 0.61(0.60,0.62) |
| Republic of Vanuatu | 10.76(-0.92,30.39) | 151.87(-13.06,432.11) | 42.14(-3.88,117.15) | 213.97(-19.43,594.27) | 1.12(1.10,1.14) | 0.78(-0.06,2.11) | 11.76(-0.93,32.31) | 2.95(-0.25,8.09) | 15.9(-1.34,43.55) | 1.00(0.97,1.02) | 9.98(-0.87,28.35) | 140.12(-12.17,400.72) | 39.19(-3.63,108.74) | 198.06(-18.10,549.83) | 1.13(1.10,1.15) |
| Republic of Yemen | 542.52(-40.62,1618.9) | 98.77(-7.35,294.55) | 2719.17(-201.17,7732.78) | 175.33(-12.76,498.89) | 1.95(1.86,2.04) | 27.3(-1.9,79.05) | 5.33(-0.36,15.47) | 156.07(-11.29,428.58) | 10.66(-0.76,29.32) | 2.37(2.30,2.45) | 515.21(-38.83,1532.43) | 93.43(-7.00,277.81) | 2563.1(-190.4,7279.34) | 164.67(-12.04,468.36) | 1.92(1.84,2.01) |
| Republic of Zambia | 282.4(-22.74,842.62) | 90.39(-7.28,269.58) | 1235.7(-102.68,3565.47) | 169.18(-13.97,490.91) | 2.09(2.07,2.11) | 24.52(-1.88,68.81) | 8.28(-0.64,23.24) | 109.55(-8.16,311.77) | 15.79(-1.18,45.19) | 2.12(2.06,2.18) | 257.88(-20.87,773.56) | 82.11(-6.63,246.43) | 1126.14(-94.26,3237.29) | 153.40(-12.74,443.59) | 2.08(2.07,2.10) |
| Republic of Zimbabwe | 569.98(-43.75,1660.76) | 118.46(-9.1,346.75) | 1582.67(-136.25,4415.97) | 201.71(-16.98,566.03) | 1.71(1.66,1.76) | 51.07(-3.65,149.5) | 11.08(-0.79,32.43) | 138.96(-11.23,381.56) | 18.88(-1.49,52.11) | 1.66(1.59,1.72) | 518.9(-39.93,1510.08) | 107.38(-8.27,314.36) | 1443.71(-123.92,3998.65) | 182.83(-15.38,509.98) | 1.71(1.66,1.76) |
| Romania | 7012.51(-592.57,19558.93) | 196(-16.55,548.56) | 12587.38(-1243.84,34263.52) | 248.8(-24.69,676.91) | 0.80(0.77,0.82) | 890.32(-74.08,2425.34) | 26.24(-2.18,71.6) | 1798.13(-162.32,4800.96) | 35.13(-3.19,93.62) | 1.00(0.97,1.03) | 6122.19(-516.45,17036.79) | 169.75(-14.32,473.86) | 10789.25(-1070.34,29388.69) | 213.68(-21.30,581.83) | 0.76(0.74,0.79) |
| Russian Federation | 49025.06(-4585.15,135748.49) | 211.72(-19.5,588.13) | 90592.66(-9138.72,238621.35) | 276.69(-27.86,728.36) | 0.94(0.91,0.97) | 5853.67(-510.09,15830.88) | 26.44(-2.27,71.79) | 12195.19(-1166.78,31763.06) | 37.8(-3.6,98.51) | 1.26(1.22,1.30) | 43171.39(-4052.8,119763.1) | 185.28(-17.15,514.86) | 78397.47(-7942.79,208178.76) | 238.89(-24.17,633.87) | 0.89(0.86,0.92) |
| Saint Kitts and Nevis | 12.15(-1.11,34.76) | 226.87(-21.07,647.63) | 28.03(-3.09,75.01) | 317.79(-34.1,852.83) | 1.10(1.06,1.13) | 0.88(-0.08,2.4) | 16.53(-1.47,45.52) | 2.09(-0.22,5.48) | 24.84(-2.5,65.58) | 1.33(1.29,1.38) | 11.27(-1.03,32.27) | 210.33(-19.52,600.55) | 25.94(-2.86,69.16) | 292.95(-31.43,783.13) | 1.08(1.04,1.11) |
| Saint Lucia | 22.06(-1.98,62.7) | 193.33(-17.36,549.86) | 83(-7.61,232.88) | 275.44(-25.21,773.69) | 1.14(1.11,1.17) | 1.51(-0.12,4.23) | 13.45(-1.06,37.92) | 6.22(-0.56,16.8) | 20.78(-1.87,56.2) | 1.41(1.36,1.45) | 20.56(-1.87,58.39) | 179.88(-16.31,511.08) | 76.79(-7,215.66) | 254.65(-23.18,715.99) | 1.12(1.10,1.15) |
| Saint Vincent and the Grenadines | 16.15(-1.31,47.24) | 173.09(-14,506.46) | 46.76(-4.17,131.77) | 251.88(-22.3,711.18) | 1.27(1.24,1.29) | 1.07(-0.08,3.07) | 11.68(-0.87,33.41) | 3.45(-0.3,9.39) | 18.98(-1.64,51.64) | 1.65(1.61,1.69) | 15.07(-1.23,44.25) | 161.41(-13.12,473.82) | 43.3(-3.87,121.88) | 232.90(-20.62,656.10) | 1.24(1.22,1.26) |
| Slovak Republic | 1792.03(-178.08,4913.31) | 228.7(-22.56,628.18) | 3430.64(-351.5,9116.48) | 265.83(-27.16,706.85) | 0.46(0.45,0.48) | 237(-22.19,626.93) | 31.05(-2.88,82.18) | 479.3(-46.18,1264.13) | 37.54(-3.61,98.99) | 0.57(0.54,0.60) | 1555.03(-155.19,4254.09) | 197.65(-19.61,541.46) | 2951.34(-302.87,7843.34) | 228.29(-23.37,606.43) | 0.45(0.43,0.46) |
| Socialist Republic of Viet Nam | 1662.7(-132.04,5074.68) | 33.23(-2.6,101.28) | 8366.24(-586.57,24684.51) | 68.02(-4.73,201.71) | 2.59(2.47,2.72) | 119.19(-8.09,349.82) | 2.45(-0.17,7.18) | 586.48(-38.81,1724.83) | 4.95(-0.32,14.57) | 2.52(2.38,2.65) | 1543.51(-124.15,4722.68) | 30.78(-2.44,94.09) | 7779.76(-547.64,22915.85) | 63.06(-4.41,186.68) | 2.60(2.47,2.72) |
| Solomon Islands | 22.78(-1.97,64.72) | 144.96(-12.46,410.33) | 75.86(-7.31,209.11) | 204.13(-19.28,564.96) | 1.08(1.02,1.14) | 1.62(-0.14,4.59) | 11.21(-0.93,31.78) | 5.4(-0.48,14.56) | 15.41(-1.35,41.77) | 0.95(0.84,1.05) | 21.16(-1.83,60.09) | 133.75(-11.51,379.25) | 70.47(-6.84,195.14) | 188.72(-17.93,524.27) | 1.09(1.03,1.15) |
| State of Eritrea | 53.51(-4.06,160.72) | 48.95(-3.59,146.38) | 242.2(-17.58,738.11) | 83.79(-6.09,255.25) | 1.72(1.67,1.78) | 3.92(-0.26,12.21) | 3.93(-0.25,12.18) | 19.3(-1.34,55.57) | 7.16(-0.5,20.58) | 1.95(1.90,2.00) | 49.59(-3.8,148.61) | 45.03(-3.33,134.56) | 222.9(-16.2,682.21) | 76.63(-5.57,234.63) | 1.70(1.64,1.76) |
| State of Israel | 1713.1(-167.48,4739.54) | 266.01(-26.05,735.34) | 5190.69(-503.87,14103.03) | 323.05(-31.51,876.73) | 0.57(0.53,0.62) | 229.21(-21.73,621.11) | 35.7(-3.39,96.72) | 751.41(-70.57,1998.71) | 46.51(-4.39,123.68) | 0.79(0.72,0.86) | 1483.89(-145.43,4142.94) | 230.31(-22.60,642.58) | 4439.28(-435.1,12034.77) | 276.54(-27.25,749.09) | 0.54(0.50,0.58) |
| State of Kuwait | 135.78(-14.05,364.01) | 238.05(-24.39,641.51) | 954.12(-119.28,2419.56) | 343.27(-41.68,875.6) | 1.25(1.23,1.27) | 9.28(-0.91,24.48) | 17.03(-1.67,44.91) | 74.32(-8.69,189.23) | 27.5(-3.15,69.76) | 1.64(1.59,1.69) | 126.5(-13.11,338.59) | 221.02(-22.67,594.36) | 879.8(-110.38,2232.78) | 315.77(-38.48,805.84) | 1.22(1.20,1.24) |
| State of Libya | 440.09(-41.84,1229.3) | 204.76(-19.09,573.28) | 1653.55(-195.78,4230.1) | 303.65(-35.57,777.44) | 1.29(1.27,1.31) | 28.44(-2.66,77.38) | 13.52(-1.24,36.8) | 115.37(-13.21,299.5) | 21.83(-2.47,56.53) | 1.58(1.53,1.63) | 411.65(-39.1,1154.54) | 191.24(-17.81,537.63) | 1538.18(-182.5,3930.01) | 281.82(-33.10,720.82) | 1.27(1.25,1.29) |
| State of Qatar | 19.92(-2.1,54.36) | 237.81(-23.71,654.03) | 252.71(-31.66,652.68) | 336.13(-39.81,877.75) | 1.09(1.06,1.12) | 1.38(-0.13,3.62) | 17.58(-1.59,46.45) | 19.16(-2.47,49.05) | 27.64(-3.31,70.66) | 1.41(1.35,1.47) | 18.54(-1.96,50.58) | 220.24(-22.06,603.99) | 233.55(-29.18,601.11) | 308.49(-36.45,802.54) | 1.06(1.04,1.09) |
| Sultanate of Oman | 107.86(-9.05,312.59) | 152.57(-12.67,442.87) | 574.48(-68.54,1517.46) | 295.96(-33.83,786.52) | 2.28(2.24,2.32) | 6.09(-0.51,16.93) | 9.02(-0.75,25.08) | 39.58(-4.34,102.61) | 21.6(-2.24,56.02) | 3.00(2.93,3.06) | 101.77(-8.53,293.25) | 143.55(-11.91,413.98) | 534.91(-64.07,1413.61) | 274.35(-31.53,728.66) | 2.23(2.20,2.27) |
| Swiss Confederation | 3214.21(-273.46,9048.39) | 238.31(-20.32,671.1) | 6346.59(-540.61,17672.55) | 274.9(-23.64,763.35) | 0.48(0.45,0.50) | 452.28(-38.93,1225.59) | 33.39(-2.88,90.53) | 948.51(-81.45,2563.11) | 40.67(-3.51,109.91) | 0.66(0.63,0.69) | 2761.93(-234.17,7827.31) | 204.92(-17.41,581.33) | 5398.08(-457.72,15107.22) | 234.23(-20.06,654.02) | 0.45(0.42,0.47) |
| Syrian Arab Republic | 1186.37(-115.31,3276.45) | 200.59(-19.32,556.1) | 4702.34(-555.12,12214.84) | 298.69(-34.32,775.19) | 1.29(1.25,1.32) | 72.17(-6.52,193.29) | 12.69(-1.14,34.25) | 306.29(-33.57,780.47) | 20.73(-2.2,53.01) | 1.54(1.48,1.60) | 1114.2(-108.95,3088.55) | 187.90(-18.20,522.73) | 4396.04(-521.32,11451.09) | 277.96(-32.10,723.45) | 1.27(1.24,1.30) |
| Taiwan (Province of China) | 2965.13(-252.53,8611.72) | 146.76(-12.31,426.61) | 14488.42(-1361.84,41190.84) | 254.12(-23.82,722.43) | 1.84(1.80,1.88) | 157.25(-12.35,446.22) | 8.19(-0.63,23.25) | 760.3(-68.33,2120.34) | 13.39(-1.2,37.4) | 1.71(1.66,1.77) | 2807.88(-240.23,8205.7) | 138.57(-11.68,404.77) | 13728.12(-1293.62,39068.97) | 240.73(-22.62,684.94) | 1.85(1.81,1.89) |
| Togolese Republic | 157.42(-11.47,463.69) | 117.54(-8.64,345.43) | 753.54(-66.11,2203.91) | 185.91(-15.82,543.22) | 1.48(1.47,1.49) | 11.57(-0.78,32.95) | 9.15(-0.62,26.14) | 57.57(-4.14,162.03) | 15.22(-1.08,42.93) | 1.63(1.60,1.66) | 145.85(-10.68,427.22) | 108.38(-8.01,316.77) | 695.96(-61.83,2034.31) | 170.69(-14.69,499.12) | 1.47(1.45,1.48) |
| Tokelau | 0.4(-0.04,1.1) | 208.28(-18.95,574.79) | 0.57(-0.06,1.49) | 296.5(-32.99,773.74) | 1.13(1.07,1.20) | 0.03(0,0.08) | 16.81(-1.44,45.67) | 0.05(0,0.13) | 24.88(-2.58,65.48) | 1.21(1.12,1.31) | 0.37(-0.03,1.02) | 191.47(-17.47,527.49) | 0.52(-0.06,1.38) | 271.62(-30.34,712.46) | 1.13(1.06,1.19) |
| Turkmenistan | 369.19(-36.18,1022.63) | 167.4(-16.11,466.44) | 923.62(-99.53,2484.74) | 201(-20.67,544.37) | 0.60(0.58,0.62) | 50.65(-4.57,137.06) | 24.01(-2.13,65.05) | 136.92(-13.3,367.43) | 30.96(-2.88,83.34) | 0.83(0.80,0.86) | 318.54(-31.56,888.55) | 143.39(-13.95,402.21) | 786.7(-85.23,2131.55) | 170.04(-17.61,462.24) | 0.56(0.55,0.58) |
| Tuvalu | 1.75(-0.16,4.88) | 200.76(-18.42,559.18) | 3.8(-0.42,10.22) | 283.92(-30.9,764.37) | 1.12(1.08,1.16) | 0.12(-0.01,0.33) | 15.04(-1.34,40.57) | 0.29(-0.03,0.76) | 22.47(-2.28,59.56) | 1.21(1.11,1.30) | 1.63(-0.15,4.56) | 185.72(-17.09,520.45) | 3.51(-0.39,9.43) | 261.45(-28.57,703.41) | 1.12(1.08,1.15) |
| Ukraine | 20558.31(-1866.28,56918.02) | 217.34(-19.34,603.28) | 27979.51(-2899.27,74829.73) | 264.65(-27.36,707.74) | 0.70(0.67,0.72) | 2522.19(-208.12,6698.09) | 27.71(-2.25,73.87) | 3692.67(-345.53,9711.46) | 35.25(-3.3,92.72) | 0.85(0.82,0.87) | 18036.12(-1652.29,49088.18) | 189.63(-17.05,516.58) | 24286.84(-2521.57,65457.09) | 229.40(-23.77,618.00) | 0.68(0.65,0.70) |
| Union of the Comoros | 21.31(-1.68,61.26) | 95.26(-7.48,274.67) | 95.43(-7.51,274.82) | 170.86(-13.36,490.74) | 1.96(1.94,1.97) | 1.84(-0.14,5.13) | 8.73(-0.65,24.45) | 8.78(-0.64,24.31) | 16.34(-1.18,45.3) | 2.10(2.08,2.13) | 19.47(-1.55,55.69) | 86.53(-6.84,248.40) | 86.65(-6.86,250.99) | 154.52(-12.16,446.97) | 1.94(1.93,1.96) |
| United Arab Emirates | 65.09(-5.54,187.04) | 191.69(-16,550.87) | 990.68(-111.41,2540.09) | 306.05(-34.44,795.77) | 1.57(1.53,1.60) | 4.09(-0.33,11.12) | 12.8(-1,35.09) | 71.52(-8.04,183.45) | 25.08(-2.76,64.52) | 2.26(2.22,2.29) | 60.99(-5.22,175.46) | 178.89(-15.00,514.82) | 919.16(-103.31,2388.8) | 280.98(-31.65,738.59) | 1.51(1.47,1.55) |
| United Kingdom of Great Britain and Northern Ireland | 36505.69(-3431.45,99847.61) | 301.04(-28.51,822.8) | 61453.98(-6370.19,163390.93) | 367.25(-38.54,976.64) | 0.69(0.60,0.77) | 5210.05(-491.01,14086.39) | 42.94(-4.06,115.96) | 9517.45(-979.92,25052.76) | 56.46(-5.86,148.16) | 0.91(0.85,0.96) | 31295.64(-2925.66,86103.29) | 258.10(-24.32,709.49) | 51936.53(-5351.62,138453.48) | 310.79(-32.42,829.14) | 0.65(0.56,0.74) |
| United Mexican States | 12292.08(-1138.67,34373.82) | 248.81(-22.86,696.34) | 51126.9(-5502.96,136862.07) | 330.51(-35.25,885.69) | 0.88(0.86,0.90) | 865.56(-77.17,2325.9) | 18.02(-1.59,48.53) | 3788.75(-393.04,9832.43) | 24.92(-2.55,64.75) | 0.80(0.65,0.95) | 11426.52(-1060.38,31903.38) | 230.79(-21.24,644.64) | 47338.15(-5106.37,126953.5) | 305.59(-32.67,820.40) | 0.89(0.87,0.90) |
| United Republic of Tanzania | 1370.65(-108.07,4041.75) | 108.05(-8.45,317.84) | 5124.73(-409.12,14647.41) | 182.79(-14.37,524.67) | 1.78(1.74,1.81) | 117.16(-8.6,338.58) | 9.81(-0.71,28.23) | 472.57(-35.34,1336.35) | 17.59(-1.3,49.62) | 1.98(1.95,2.00) | 1253.49(-99.19,3703.91) | 98.24(-7.72,289.28) | 4652.15(-373.68,13324.12) | 165.20(-13.06,474.66) | 1.76(1.72,1.79) |
| United States of America | 140696.39(-13502.38,382713.69) | 332.9(-32.06,904.98) | 323868.94(-34953.65,854278.45) | 410.85(-44.47,1083.52) | 0.39(0.22,0.56) | 21676.94(-2141.38,60082.88) | 51.18(-5.06,141.77) | 53126.95(-5770.65,137078.97) | 67.23(-7.31,173.39) | 1.04(0.93,1.15) | 119019.45(-11324.45,324467.95) | 281.72(-26.90,767.97) | 270742(-29025.09,722755.01) | 343.62(-36.94,917.26) | 0.26(0.06,0.45) |
| United States Virgin Islands | 29.71(-3.08,81.6) | 304.83(-31.2,838.49) | 91.9(-9.35,248.74) | 360.6(-36.79,974.83) | 0.53(0.51,0.56) | 2.31(-0.22,6.17) | 24.48(-2.28,65.5) | 7.65(-0.74,20.09) | 30.15(-2.92,79.24) | 0.67(0.63,0.72) | 27.4(-2.86,75.7) | 280.35(-28.85,774.50) | 84.25(-8.6,228.92) | 330.44(-33.81,896.34) | 0.52(0.49,0.54) |

ASYR: Age-standardized Years lived with disability rate; YLDs: Years Lived with Disability; EAPC: Estimated Average Percentage Change.
